# Women's groups, covariate shocks, and resilience: An evidence synthesis of past shocks to inform a response to COVID-19

**DOI:** 10.12688/gatesopenres.14771.1

**Published:** 2023-07-20

**Authors:** Rebecca Walcott, Carly Schmidt, Marina Kaminsky, Roopal Jyoti Singh, Leigh Anderson, Sapna Desai, Thomas de Hoop

**Affiliations:** 1 American Institutes for Research, Arlington, Virginia, USA; 2 State of Washington, Olympia, WA, USA; 3 North Star Civic Foundation, Portland, OR, USA; 4 Population Council Institute, New Delhi, India; 5 University of Washington, Seattle, Washington, USA; 6 Population Council India, New Delhi, Delhi, India

**Keywords:** Women’s groups, Covariate shock, Resilience, Evidence synthesis, COVID-19

## Abstract

*Background:* Interventions with women’s groups are increasingly seen as an important strategy for advancing women’s empowerment, health, and economic outcomes in low- and middle-income countries, with the potential to increase the resiliency of members and their communities during widespread covariate shocks, such as coronavirus disease 2019 (COVID-19).

*Methods:* This evidence synthesis compiles evidence from past shocks on women’s group activities and the extent to which women’s groups mitigate the effects of shocks on members and communities. We reviewed 90 documents from academic databases, organizational reports, and additional gray literature, and included literature diverse in geography, type of women’s group, and shock.

*Results:* The literature suggests that covariate shocks tend to disrupt group activities and reduce group resources, but linkages to formal institutions can mitigate this impact by extending credit beyond the shock-affected resource pool. Evidence was largely supportive of women’s groups providing resilience to members and communities, though findings varied according to shock severity, group purpose and structure, and outcome measures. Further, actions to support individual resilience during a shock, such as increased payment flexibility, may run counter to group resilience. The findings of the evidence synthesis are largely consistent with emerging evidence about women’s groups and COVID-19 in South Asia and sub-Saharan Africa.

*Conclusions:* We finalize the paper with a discussion on policy implications, including the importance of sustainable access to financial resources for women’s group members; equity considerations surrounding the distribution of group benefits and burdens; and the potential for meaningful partnerships between women’s groups and local governments and/or non-governmental organizations (NGOs) to enhance community response amidst crises.

## Introduction

Interventions with women’s groups are increasingly recognized as an important strategy for advancing women’s empowerment, health, and economic outcomes in low- and middle-income countries (LMICs). *Women’s groups* is an umbrella term commonly used to refer to different models of groups whose membership is primarily female. Objectives of women’s groups may include promoting financial inclusion and women’s economic empowerment, organizing workers in the informal sector, and improving health outcomes among group members and their communities.

The coronavirus disease 2019 (COVID-19) crisis has highlighted that while acute covariate shocks pose unique challenges for women and women’s groups, women’s groups may provide members and their communities with tools to increase their resiliency to crises ( Adegbite *et al.,* 2022; de Hoop *et al.,* 2020). ^1^ For example, women’s groups can offer an immediate support network for members through mechanisms such as pooled resources and social capital. Groups can adapt their in-place human infrastructure to play a key role in community responses to shocks by disseminating important information, contributing to crisis responses by providing necessary household and community services, and helping members obtain access to social protection. In the case of COVID-19, Indian self-help groups (SHGs) and savings groups in sub-Saharan Africa also contributed to producing masks, operating community kitchens, and running help desks ( Adegbite *et al.,* 2022; de Hoop *et al.,* 2020; de Hoop *et al.,* 2021).

This evidence synthesis contributes to the existing literature by compiling evidence of how shocks affected women’s groups and their members prior to the COVID-19 crisis. We first examine how these shocks affected the implementation and activities of women’s groups in LMICs. Next, we examine the extent to which women’s groups promoted resilience for their members and communities. Previous evidence from systematic reviews and impact evaluations suggests that women’s groups with economic objectives, such as SHGs in India and savings groups in sub-Saharan Africa, can achieve positive effects on women’s empowerment and economic outcomes ( *e.g.,* Barooah *et al.,* 2020; Brody *et al.,* 2017). However, it is unclear whether these findings can be extrapolated to contexts experiencing covariate shocks or to the COVID-19 crisis in particular because social distancing may limit the opportunities of groups to meet ( de Hoop *et al.,* 2020).

Overall, the evidence revealed that covariate shocks tend to disrupt group activities and reduce group resources, but women’s groups can support the resilience of members and communities - though findings varied across contexts. However, for economic groups in particular, this improved resilience may come at the expense of group resources, indicating a trade-off between member resilience and group sustainability. For example, actions to support individual resilience during a shock, such as increased payment flexibility, may threaten group sustainability by depleting group resources. The evidence further indicates that linkages to formal institutions can mitigate negative effects on group resilience by providing credit beyond the shock-affected resource pool. These findings are largely consistent with emerging evidence on women’s groups and COVID-19 ( Adegbite *et al.,* 2022; Agarwal, 2021; de Hoop *et al.*, 2021; Siwach *et al.*, 2023), indicating that this evidence synthesis can provide important lessons for policy responses to COVID-19 in South Asia and sub-Saharan Africa. 

### Women’s groups and resilience

There are many different models of women’s groups, and they vary in terms of organizing objective, function, size, governance, and linkages to formal institutions ( Desai *et al.,* 2022). Groups with economic objectives, such as SHGs, village savings and loan associations (VSLAs), and rotating credit and savings associations (ROSCAs), are formed to promote savings and pool funds from which members can borrow. Other types of groups may focus on health, agriculture, advocacy, or community resource management – or a particular population group such as mothers, sexual violence survivors, or adolescent girls. Women’s groups may be standalone village or urban groups or regional network associations of groups. They may operate independently or with the support of non-governmental organizations (NGOs) and/or formal financial institutions.

This broad definition of women’s groups enables an inclusive review of the evidence, but such breadth may mask important differences that limit cross-group comparisons. For example, a savings group might play a very different role than a community resource management group when responding to a particular shock. However, despite differing in function and form, most women’s groups, even as broadly defined, share some characteristics that allow for examining variation in the relationship of women’s groups to shocks across contexts.

We conceptualize resilience, our primary outcome of interest, along three dimensions: an *absorptive capacity* that refers to the ability to cope with or absorb shocks; an *adaptive capacity* that includes learning and strategic adjustments to mitigate the effects of shocks; and a *transformative capacity* that involves a systemic adjustment to the status quo that reduces vulnerability to shocks ( Bene *et al.,* 2015; Tanner *et al.,* 2017; Vaughan & Frankenberger, 2018). Individual resilience can refer to individual coping mechanisms and adjustments in various domains, including food security, consumption smoothing and savings, income and employment, psychological outcomes, and health outcomes. Group resilience can refer to the short-term ability of the group to continue functioning as before the shock or to the group’s ability to adapt to the shock, and to the longer-term ability of the group to continue functioning or group sustainability. There are many theorized mechanisms through which women’s groups can enhance resilience for members in the face of covariate shocks ( CARE, 2015; Diaz-Martin *et al.,* 2023; Gram *et al.,* 2019; Vaughan & Frankenberger, 2018; Weingärtner *et al.,* 2017). Financial mechanisms that serve to increase members’ absorptive capacity include pooling risk and resources, social commitment to savings, and potential access to formal financial services through group linkages. For example, a VSLA or SHG may provide members with savings and credit with which to absorb the economic losses resulting from a rainfall shock ( Demont, 2013; Karlan *et al.,* 2017). Social support mechanisms may enhance members’ psychological resilience to shocks ( Berry, 2015; Tol *et al.,* 2020). Some groups facilitate learning and technical skills, such as water, sanitation, and hygiene training, and this access to new information may strengthen members’ adaptive capacity when faced with waterborne disease outbreaks ( Khatibi *et al.,* 2011). Similarly, groups provide access to information networks that enable members to adopt livelihood diversification strategies to smooth household income during shocks ( Demont, 2013). Mechanisms such as collective action and group bargaining power can contribute to transformative capacity when women’s groups mobilize for system-level change, such as for more inclusive disaster response policy ( Clissold *et al.,* 2020; Fordham *et al.,* 2011).

Our examination of both individual and group resilience highlights the existence of a possible tension between strategies that promote the resilience of groups ( *i.e.,* the group functioning and infrastructure withstanding the shock) and the resilience of group members ( *i.e.,* individual group members withstanding the shock). While the three resilience capacities discussed above (absorptive, adaptive and transformative capacity) offer a helpful background framework for categorising individual resilience, we find that evidence is largely reported in terms of absorptive capacity for individuals; that is, the literature on women’s groups and resilience tends to prioritize individual or household outcome measures of consumption smoothing attributable to pooled resources and access to credit. We also find a lack of discussion around mechanisms of group resilience, particularly in the case of aggregate shocks that may threaten group sustainability. In our discussion of the evidence, we distinguish between results related to the ways in which covariate shocks may affect women’s groups and how groups may adapt policies or activities to sustain *group* resilience (in Section *How do Shocks Affect Women’s Groups?*) and results examining evidence pertaining to *individual* (or household) resilience (in Section *How Do Women’s Groups Mitigate the Effects of Shocks for Members?*).

This review was motivated by the COVID-19 crisis, and accordingly focuses specifically on member and group resilience to acute covariate shocks. These shocks include natural disasters, disease outbreaks, conflict, economic crises, and other events that have the potential to negatively affect all members of a group simultaneously. Because of the focus on mechanisms specific to covariate shocks, such as natural disasters or COVID-19, we did not examine group members’ resilience to idiosyncratic shocks that affect members at the individual level, such as the death of a household member, or to chronic stressors such as poverty or endemic disease.

## Methods

We examine both immediate and longer-term effects of covariate shocks on women’s groups and their members. In this way we add to existing evidence about the short-term effects of COVID-19 on SHG savings in India ( Siwach *et al.*, 2023) and how SHGs in India and savings groups in Nigeria may have contributed to mitigating some of the negative short-term effects of COVID-19 on agricultural productivity, consumption, and food security ( Adegbite *et al.,* 2022; Agarwal, 2021; de Hoop *et al.,* 2021). Triangulating the findings from those studies with the findings from the evidence synthesis allows for learning about the potential longer-term effects of COVID-19 on women’s groups and their members. Our evidence synthesis is derived from academic databases, organizational reports, and additional gray literature. Using a targeted search strategy, we extracted 90 total documents from over 2800 search results.

We conducted an evidence synthesis of the literature on women’s groups and acute covariate shocks with three primary research questions in mind:

i. How do shocks affect women’s groups? That is, to what extent do shocks affect group resources and activities; how do women’s groups adapt to shocks; and what implementation features contribute to group resilience during shocks?
ii. How do women’s groups mitigate the effects of shocks for members? That is, to what extent do women’s groups increase the resilience of their members to shocks; what is the variation by shock, group type, and mechanism?
iii. How do women’s groups support community responses to shocks?

Our search strategy aimed to identify, consolidate, and synthesize existing evidence on the impact of shocks on women’s groups and the ability of women’s groups to mitigate the effects of shocks for their members and their communities. We searched both peer-reviewed literature in academic databases and gray literature for case studies, evaluations, and other evidence on women’s groups and shocks. We searched the following databases for literature in English:

- Academic databases (peer-reviewed and gray literature): Google Scholar, Scopus, PubMed, EconLit, Embase, PAIS
- Organization websites and databases: UN Development Fund for Women; BRAC Institutional Repository; World Bank E-Library; Population Council; CARE; Women for Women International; Africa Portal Research Library; Evidence Consortium on Women’s Groups Evidence Repository; 3ie Evidence Hub; WHO IRIS; India’s National Rural Livelihoods Mission

We constructed search strings from keyword components for women’s groups and a variety of shocks. We incorporated both general and highly specific search strings to return a comprehensive list of search results. We employed search strings in both the academic databases and organization websites listed above and supplemented our results with targeted Google searches and forward/backward searching within the reference lists of relevant articles.

Our search process included academic databases of peer-reviewed articles as well as targeted searches of organizational reports, news articles, blog posts, and additional gray literature. From June to August 2020, we reviewed over 2800 search results and included 90 documents that: i) met our definitions of women’s groups and shocks, ii) were set in a LMIC and published in 1999 or later, iii) were written in English, and iv) addressed at least one of our research questions. After building the sample of included literature to review, we extracted information on the document type, geography, women’s group, shock, and content relevant to our research questions using a coding spreadsheet. Empirical studies were coded in Microsoft Excel according to research design and methodology, but we did not apply a comprehensive risk of bias assessment to appraise the quality of the evidence. The coding spreadsheet is available in Table 1.

**Table 1.** Included evidence (n=90).

Citation	Document type	Study type	Region	Country	Women's group	Shock
Ahmed, D. (1999). Vegetable growers of BRAC and flood 1998: a case study of a village organization in Gaibandha district. Experiences of Deluge: Flood 1998, 77.	Organization Report	Qualitative	South Asia	Bangladesh	Microfinance Group	Natural - Bangladesh floods 1998
Alam, K., & Rahman, M. H. (2017). The role of women in disaster resilience. Handbook of disaster risk reduction and management. World Scientific Press, New Jersey, 697–719.	Book Chapter	Review	Multiple	India, Jamaica, Japan, Nicaragua, Mexico	Unspecified	Natural - Various
Anaandan, S. (2018). Kerala floods: Kudumbashree women play big role in clean-up. The Hindu.	News Article	Qualitative	South Asia	India	Self-Help Group	Natural - Kerala Floods 2018
Androsik, A. (2020). Gendered understanding of ebola crisis in Sierra Leone. Lessons for covid-19. Population and Economics, 4(2), 88–95.	Peer-reviewed Journal Article	Mixed Methods	Sub-Saharan Africa	Sierra Leone	Other Savings Group	Health - Ebola
Ashraf, N., Giné, X., & Karlan, D. (2009). Finding missing markets (and a disturbing epilogue): Evidence from an export crop adoption and marketing intervention in Kenya. American Journal of Agricultural Economics, 91(4), 973–990.	Peer-reviewed Journal Article	Quantitative	Sub-Saharan Africa	Kenya	Self-Help Group	Economic - Change in export policy
Atela, J., Gannon, K. E. and Crick, F. (2018). Climate change adaptation among female-led micro, small and medium enterprises in semi- arid areas: A case study from Kenya.’, in Leal Filho, W. (ed.) Handbook of Climate Change Resilience. Cham: Springer, pp. 1–18.	Book Chapter	Qualitative	Sub-Saharan Africa	Kenya	Other Savings Group	Natural - Climate shocks
Bahadur, A., Lovell, E., & Pichon, F. (2016). Effectiveness in building resilience: Synthesis report for Oxfam's resilience outcome area. Oxfam Research Reports.	Organization Report	Review	Sub-Saharan Africa	Senegal	Other Savings Group	Natural - Climate amd weather shocks
Bandiera, O., Buehren, N., Goldstein, M. P., Rasul, I., & Smurra, A. (2019). The Economic lives of young women in the time of ebola: Lessons from an empowerment program. The World Bank.	Working Paper	Quantitative	Sub-Saharan Africa	Sierra Leone	ELA and other adolescent girls groups	Health - Ebola
Bandiera, O., Buehren, N., Goldstein, M., Rasul, I., & Smurra, A. (2020). Do school closures during an epidemic have persistent effects? Evidence from Sierra Leone in the time of ebola. Working Paper - Poverty Action Lab.	Working Paper	Quantitative	Sub-Saharan Africa	Sierra Leone	ELA and other adolescent girls groups	Health - Ebola
BARA & IPA. (2013). Final impact evaluation of the saving for change program in Mali, 2009–2012.	Organization Report	Mixed Methods	Sub-Saharan Africa	Mali	Other Savings Group	Multiple - lean season, drought, livestock disease, conflict
Bass, J., Murray, S., Cole, G., Bolton, P., Poulton, C., Robinette, K., … Annan, J. (2016). Economic, social and mental health impacts of an economic intervention for female sexual violence survivors in Eastern Democratic Republic of Congo. Global Mental Health, 3(19).	Peer-reviewed Journal Article	Quantitative	Sub-Saharan Africa	DRC	VSLA	Conflict - Persistent conflict and mass sexual violence
Benni, N., & Barkataky, R. (2018). The role of the Self Employed Women’s Association (SEWA) in providing financial services to rural women. FAO.	Organization Report	Qualitative	South Asia	India	Self-Help Group	Natural - Earthquake
Bermudez, L., & Matuszeski, J. (2010). Ensuring continued success: Saving for change in older program areas of Mali. Oxfam America.	Organization Report	Qualitative	Sub-Saharan Africa	Mali	Other Savings Group	Natural - Rainfall shock
Berry, M. E. (2015). From violence to mobilization: Women, war, and threat in Rwanda. Mobilization: An International Quarterly, 20(2), 135–156.	Peer-reviewed Journal Article	Qualitative	Sub-Saharan Africa	Rwanda	Self-Help Group	Conflict - Genocide
Branco, A. de M. (2009). Women responding to drought in Brazil. In Women, Gender and Disaster: Global Issues and Initiatives (pp. 261–272). SAGE Publications India Pvt Ltd.	Book Chapter	Qualitative	Latin America and Caribbean	Brazil	Collective Action and Grassroots Group	Natural - Drought
Brickman Raredon, A. (2011). Opportunity in Haiti: Women as agents of resilience in post-disaster reconstruction [Thesis, Massachusetts Institute of Technology].	Thesis	Mixed Methods	Latin America and Caribbean	Haiti	Multiple	Natural - Earthquake
Brownhill, L. (2009). A climate for change: Humanitarian disaster and the movement for the commons in Kenya. In Women, Gender and Disaster: Global Issues and Initiatives (pp. 224–232). SAGE Publications India Pvt Ltd.	Book Chapter	Qualitative	Sub-Saharan Africa	Kenya	Multiple	Conflict - Social Unrest
Brunie, A., Fumagalli, L., Martin, T., Field, S., & Rutherford, D. (2014). Can village savings and loan groups be a potential tool in the malnutrition fight? Mixed method findings from Mozambique. Children and Youth Services Review, 47, 113–120.	Peer-reviewed Journal Article	Mixed Methods	Sub-Saharan Africa	Mozambique	Multiple	Natural - Hunger Season
Buehren, N., Chakravarty, S., Goldstein, M., Slavchevska, V., & Sulaiman, M. (2017). Adolescent girls’ empowerment in conflict- affected settings: Experimental evidence from South Sudan. CSAE Conference Paper.	Conference Paper	Quantitative	Sub-Saharan Africa	South Sudan	ELA and other adolescent girls groups	Conflict - Ethnic conflict
Camara, S., Delamou, A., Millimouno, T. M., Kourouma, K., Ndiaye, B., & Thiam, S. (2020). Community response to the Ebola outbreak: Contribution of community-based organisations and community leaders in four health districts in Guinea. Global Public Health, 1–11.	Peer-reviewed Journal Article	Mixed Methods	Sub-Saharan Africa	Guinea	Unspecified	Health - Ebola
CARE (2015). Resilience champions: when women contribute to the resilience of communities in the Sahel through savings and community-based adaptation.	Organization Report	Qualitative	Sub-Saharan Africa	Niger, Mali	VSLA	Multiple - Various
Care. (2020). Learning Brief: VSLA and CARE Adaptations to COVID-19 and Past Crises.	Organization Report	Review	Sub-Saharan Africa	Benin, Burundi, Democratic Republic of the Congo, Mozambique, Niger, Nigeria, Rwanda, Tanzania, Uganda, Haiti, Chad, Mali, Sierra Leone, Somalia, Bangladesh, Ethiopia	VSLA	Health - Covid-19, Ebola, HIV
Christian, P., Kandpal, E., Palaniswamy, N., & Rao, V. (2019). Safety nets and natural disaster mitigation: evidence from cyclone Phailin in Odisha. Climatic Change, 153(1–2), 141–164.	Peer-reviewed Journal Article	Quantitative	South Asia	India	Self-Help Group	Natural - Flood
Clissold, R., Westoby, R., & McNamara, K. E. (2020). Women as recovery enablers in the face of disasters in Vanuatu. Geoforum, 113, 101–110.	Peer-reviewed Journal Article	Qualitative	East Asia and Pacific	Vanuatu	Multiple	Natural - Cyclone
Coppock, D. L., & Desta, S. (2013). Collective action, innovation, and wealth generation among settled pastoral women in northern Kenya. Rangeland Ecology & Management, 66(1), 95–105. JSTOR.	Peer-reviewed Journal Article	Mixed Methods	Sub-Saharan Africa	Kenya	Multiple	Natural - Drought
Corbin, J. N., & Hall, J. C. (2019). Resettlement post conflict: Risk and protective factors and resilience among women in northern Uganda. International Social Work, 62(2), 918–932.	Peer-reviewed Journal Article	Qualitative	Sub-Saharan Africa	Uganda	VSLA	Conflict - Violence; displacement
Darychuk, A., & Jackson, S. (2015). Understanding community resilience through the accounts of women living in west bank refugee camps. Affilia, 30(4), 447–460.	Peer-reviewed Journal Article	Qualitative	Middle East/ North Africa (MENA)	Palestine	Other	Conflict - Violence; displacement
De, S. (2011). The whims of indian monsoons: Long-term health consequences of early childhood exposure to the indian drought of 2002. Young Lives Student Paper. Retrieved from:	Thesis	Quantitative	South Asia	India	Self-Help Group	Natural - Drought
Deepa, T. M., Rao, E. V., Patil, R. R., & Samuel, R. (2008). Operational feasibility of establishing community reporting systems. National Medical Journal of India, 21(4), 166–170.	Peer-reviewed Journal Article	Mixed Methods	South Asia	India	Self-Help Group	Health - Infectious diseases
Demont, T. (2013). Poverty, access to credit and absorption of weather shocks: evidence from Indian self-help groups. CRED working paper.	Working Paper	Quantitative	South Asia	India	Self-Help Group	Natural - Rainfall shock
Demont, T. (2022). Coping with shocks: How self-help groups impact food security and migration. World Development, 155, 105892	Peer-reviewed Journal Article	Quantitative	South Asia	India	Self-Help Group	Natural - Rainfall shock
Dumas, T. (2016). Mitigating the impact of the ebola virus disease on the most vulnerable households through an integrated food and nutrition security intervention in the district of Moyamba, Sierra Leone.	Organization Report	Qualitative	Sub-Saharan Africa	Sierra Leone	VSLA	Health - Ebola
Enarson, E. (2001). We want work: Rural women in the Gujarat drought and earthquake. Natural Hazards Research and Applications Information Center.	Working Paper	Qualitative	South Asia	India	Other Savings Group	Natural - Earthquake, drought
Enarson, E., Fothergill, A., & Peek, L. (2007). Gender and disaster: Foundations and directions. In H. Rodríguez, E. L. Quarantelli, & R. R. Dynes (Eds.), Handbook of Disaster Research (pp. 130–146). Springer.	Book Chapter	Review	Multiple	Multiple	Collective Action and Grassroots Group	Natural - Disaster
Falk, M.L. (2014) Gender and Buddhism in the wake of the tsunami. In: Liamputtong P. (eds) Contemporary Socio-Cultural and Political Perspectives in Thailand. Springer, Dordrecht.	Book Chapter	Qualitative	East Asia and Pacific	Thailand	Self-Help Group	Natural - Tsunami
Féron, É. (2020). Reinventing conflict prevention? Women and the prevention of the reemergence of conflict in Burundi. Conflict Resolution Quarterly, 37(3).	Peer-reviewed Journal Article	Qualitative	Sub-Saharan Africa	Burundi	Unspecified	Conflict - Political unrest, violence, displacement
Fisher, S. (2009). Sri Lankan women’s organisations responding to post-tsunami violence. In Women, Gender and Disaster: Global Issues and Initiatives (pp. 233–249). SAGE Publications India Pvt Ltd.	Book Chapter	Qualitative	South Asia	Sri Lanka	Unspecified	Natural - Tsunami
Fordham, M., Gupta, S., Akerkar, S., & Scharf, M. (2011). Leading resilient development: Grassroots women's priorities, practices and innovations. United Nations Development Programme (UNDP).	Organization Report	Qualitative	Multiple	Honduras, India, Philippines, Turkey, Sri Lanka	Multiple	Natural - Various (hurricane, tsunami, floods, earthquake)
Garikipati, S. (2008). The impact of lending to women on household vulnerability and women’s empowerment: Evidence from India. World Development, 36(12), 2620–2642.	Peer-reviewed Journal Article	Quantitative	South Asia	India	Self-Help Group	Natural - Drought
Gash, M., & Gray, B. (2016). The role of financial services in building household resilience in Burkina Faso. CGAP Clients at the Center.	Working Paper	Qualitative	Sub-Saharan Africa	Burkina Faso	Other Savings Group	Economic - Harvest-related economic shocks
Ghosh (2019). After cyclone Fani, women in a migrant fishing community start resilience fund. Mongabay Series: Environment and Her.	Blog	Qualitative	South Asia	India	Self-Help Group	Natural - Cyclone Fani
Govt. of Odisha, UN, World Bank, Asian Development Bank (2019). Damage, loss and needs assessment: Odisha (India).	Organization Report	Qualitative	South Asia	India	Multiple	Natural - Cyclone Fani
Gupta, S., & Leung, I. S. (2011). Turning good practice into institutional mechanisms: Investing in grassroots women's leadership to scale up local implementation of the Hyogo Framework for Action. Huairou Commission and Groots International, Brooklyn, NY.	Organization Report	Mixed Methods	Multiple	Multiple	Collective Action and Grassroots Group	Natural - General
Hedger, M., Singha, A., & Reddy, M. (2010). Building climate resilience at state level: Disaster risk management and rural livelihoods in Orissa. Strengthening Climate Resilience Discussion Paper 5.	Organization Report	Review	South Asia	India	Self-Help Group	Natural - Climate shocks
Heltberg, R., Hossain, N., Reva, A., & Turk, C. (2013). Coping and resilience during the food, fuel, and financial crises. Journal of Development Studies, 49(5), 705–718.	Peer-reviewed Journal Article	Qualitative	Multiple	Philippines, Indonesia, Senegal, CAR	Other Savings Group	Economic - The food, fuel, and financial crises during 2008– 2011
Hossain, M. Z., & Rahman, M. A. U. (2018). Pro-poor adaptation for the urban extreme poor in the context of climate change. International Journal of Climate Change Strategies and Management.	Peer-reviewed Journal Article	Qualitative	South Asia	Bangladesh	Other Savings Group	Natural - Bangladesh floods 1998
Huang, Y., & Wong, H. (2013). Effects of social group work with survivors of the Wenchuan earthquake in a transitional community. Health & Social Care in the Community, 21(3), 327–337.	Peer-reviewed Journal Article	Qualitative	East Asia and Pacific	China	Other	Natural - Earthquake
Jahns, E. (2014). Savings groups, shocks and coping strategies: The case of poor rural households in El Salvador (Doctoral dissertation, Fletcher School of Law and Diplomacy (Tufts University)).	Dissertation	Mixed Methods	Latin America and Caribbean	El Salvador	Other Savings Group	Economic - High food prices
Joshi, C., & Bhatt, M. R. (2009). Engendering tsunami recovery in Sri Lanka: The role of UNIFEM and its partners. In Women, Gender and Disaster: Global Issues and Initiatives (pp. 304–319). SAGE Publications India Pvt Ltd.	Book Chapter	Qualitative	South Asia	Sri Lanka	Unspecified	Natural - Tsunami
Kaboski, J. P., & Townsend, R. M. (2005). Policies and impact: An analysis of village-level microfinance institutions. Journal of the European Economic Association, 3(1), 1–50.	Peer-reviewed Journal Article	Quantitative	East Asia and Pacific	Thailand	Microfinance Group	Economic - Various
Karlan, D., Savonitto, B., Thuysbaert, B., & Udry, C. (2017). Impact of savings groups on the lives of the poor. Proceedings of the National Academy of Sciences, 114(12), 3079–3084.	Peer-reviewed Journal Article	Quantitative	Sub-Saharan Africa	Ghana, Malawi, Uganda	VSLA	Natural - Drought
Kellogg, M. (2020). Women building resilient cities in the context of climate change: Lessons from Freetown, Sierra Leone. Georgetown Institute for Women, Peace and Security 2020.	Organization Report	Qualitative	Sub-Saharan Africa	Sierra Leone	Other Savings Group	Natural - Flood
Khatibi, F. S., Ramalingam, A., & Yamakanamardi, S. M. (2011). Role of Women in Prevention of Epidemic Waterborne Diseases Through Training Programmes in Mysore City. Nature, Environment and Pollution Technology, 10(2), 243–246.	Peer-reviewed Journal Article	Qualitative	South Asia	India	Self-Help Group	Health - Waterborne Epidemics
Kilby, P. (2008). The strength of networks: the local NGO response to the tsunami in India. Disasters, 32(1), 120–130.	Peer-reviewed Journal Article	Qualitative	South Asia	India	Multiple	Natural - Tsunami
Korkoyah Jr, D. T., & Wreh, F. F. (2015). Ebola impact revealed: An assessment of the differing impact of the outbreak on the women and men in Liberia. Oxfam International.	Organization Report	Mixed Methods	Sub-Saharan Africa	Liberia	Other Savings Group	Health - Ebola
Kruks-Wisner, G. (2011). Seeking the local state: gender, caste, and the pursuit of public services in post-tsunami India. World Development, 39(7), 1143–1154.	Peer-reviewed Journal Article	Qualitative	South Asia	India	Self-Help Group	Natural - Tsunami
Ksoll, C., Lilleør, H. B., Lønborg, J. H., & Rasmussen, O. D. (2016). Impact of village savings and loan associations: Evidence from a cluster randomized trial. Journal of Development Economics, 120, 70–85.	Peer-reviewed Journal Article	Quantitative	Sub-Saharan Africa	Malawi	VSLA	Natural - Hunger Season
Kuppuswamy, S., & Rajarathnam, S. (2009). Women, information technology and disaster management: Tsunami affected districts of Tamil Nadu. International Journal of Innovation and Sustainable Development, 4(2/3), 206–2015.	Peer-reviewed Journal Article	Qualitative	South Asia	India	Self-Help Group	Natural - Tsunami
Langlay, N. (2014). The impact of ebola virus disease on village savings and loans associations Montserrado, Margibi, Bong and Lofa counties. FAO.	Organization Report	Mixed Methods	Sub-Saharan Africa	Liberia	VSLA	Health - Ebola
Larson, G., Drolet, J., & Samuel, M. (2013). The role of self-help groups in post-tsunami rehabilitation. International Social Work, 58(5), 732–742.	Peer-reviewed Journal Article	Qualitative	South Asia	India	Self-Help Group	Natural - Tsunami
Linkow, B., & Rentschler, L. (2016). Fraying of the ties that bind: Community-level Financial institutions and HIV/AIDS with evidence from KwaZulu-Natal, South Africa. Journal of African Economies, 1–21.	Peer-reviewed Journal Article	Quantitative	Sub-Saharan Africa	South Africa	Other Savings Group	Health - HIV/ AIDS during period of rapidly increasing mortality
LTS International. (2015). ECRP flood study: Assessing the contribution of ECRP to flood resilience.	Organization Report	Mixed Methods	Sub-Saharan Africa	Malawi	VSLA	Natural - Flood
Mehta, M. (2009). Reducing disaster risk through community resilience in the Himalayas. In Women, Gender and Disaster: Global Issues and Initiatives (pp. 57–74). SAGE Publications India Pvt Ltd.	Book Chapter	Review	South Asia	India	Unspecified	Natural - Cloudburst
Mitchell, M. (2018). The Curse of the Kosi. Heifer International.	Blog	Qualitative	South Asia	India	Self-Help Group	Natural - Floods
Moser, C., Norton, A., Stein, A., & Georgieva, S. (2010). Pro-poor adaptation to climate change in urban centers: Case studies of vulnerability and resilience in Kenya and Nicaragua. The World Bank.	Organization Report	Qualitative	Multiple	Kenya, Nicaragua	Multiple	Natural - Rainfall shocks
Mukenge, M. (2013). The role of grassroots women’s groups in HIV/AIDS prevention and response: Examples of practice in post- conflict settings. International Peacekeeping, 20(4), 469–485.	Peer-reviewed Journal Article	Qualitative	Sub-Saharan Africa	Sierra Leone, Democratic Republic of the Congo	Multiple	Health - HIV
Mukti (2020). Mukti provides aid to the SHG women group at Radhakantapur Gram Panchayat.	News Article	Qualitative	South Asia	India	Self-Help Group	Natural - Cyclone Amphan
Mulyasari, F., & Shaw, R. (2014). Risk communication through community-based society organizations as local response to disaster in Bandung, Indonesia. In Risks and conflicts: Local responses to natural disasters. Emerald Group Publishing Limited.	Book Chapter	Qualitative	East Asia and Pacific	Indonesia	Other	Natural - Disaster
Nambiar, M. (2016). The growing role of women in disaster risk management. World Bank.	Blog	Qualitative	South Asia	India	Self-Help Group	Natural - Cyclone Phailin
Nannozi, A. (2019). A case study: Exploring the influence of the informal financial sector on food security among smallholder farmers in Uganda, Greater Luweero.	Thesis	Qualitative	Sub-Saharan Africa	Uganda	VSLA; Other Savings Group	Economic - Price shocks
Nayar, N., & Faisal, M. E. H. (1999). Microfinance survives Bangladesh floods. Economic and Political Weekly, 34(14), 801–803. JSTOR.	Peer-reviewed Journal Article	Qualitative	South Asia	Bangladesh	Microfinance Group	Natural - Bangladesh floods 1998
Pollard, A. A. (2003). Women’s oganizations, voluntarism, and self- financing in Solomon Islands: a participant perspective. Oceania, 74(1–2), 44–60.	Peer-reviewed Journal Article	Qualitative	South Pacific	Solomon Islands	Multiple	Conflict - Ethnic conflict
Population Council. (2020). Covid-19 research results brief # 7: Self- help groups: A potential pivot of Bihar’s response to covid-19.	Organization Report	Qualitative	South Asia	India	Self-Help Group	Health - Covid-19
Porter, M. (2001). Women in “Reformasi” Aspects of Women's Activism in Jakarta. Canadian Journal of Development Studies/ Revue canadienne d'études du développement, 22(1), 51– 80..2001.9668802	Peer-reviewed Journal Article	Qualitative	South Asia	Indonesia	Collective Action and Grassroots Group	Conflict - Sexual violence
Ravon, L. (2014). Resilience in times of food insecurity: Reflecting on the experiences of women’s organizations. Oxfam Canada.	Organization Report	Qualitative	Multiple	Peru, Brazil, Guatemala, Nicaragua, El Salvador, South Africa, Ethiopia, Burkina Faso, Niger, Sri Lanka	Multiple	Natural - Disaster
Ray-Bennett, N. S. (2010). The role of microcredit in reducing women’s vulnerabilities to multiple disasters. Disasters, 34(1), 240–260.	Peer-reviewed Journal Article	Qualitative	Sub-Saharan Africa	India	Self-Help Group	Natural - Super cyclone
Ruszczyk, H. A. (2014). Local understandings of community resilience in earthquake prone Nepal. Durham theses, Durham University.	Dissertation	Qualitative	South Asia	Nepal	Multiple	Natural - Earthquake
Shaji, Shilpa (2020). COVID-19: Local self-governments, SHGs key to tackling pandemic in Kerala, says former chief secretary.	News Article	Qualitative	South Asia	India	Self-Help Group	Health - Covid-19
Sharma, V., Reddy, B., & Sahu, N. (2014). Sustainable rural livelihoods approach for climate change adaptation in Western Odisha, Eastern India. Development in Practice, 24(4), 591–604.	Peer-reviewed Journal Article	Qualitative	South Asia	India	Self-Help Group	Natural - Climate shocks
Sim, T., Lau, J., Cui, K., & Wei, H.-H. (2019). Post-disaster psychosocial capacity building for women in a Chinese rural village. International Journal of Disaster Risk Science, 10(2), 193–203.	Peer-reviewed Journal Article	Qualitative	East Asia and Pacific	China	Other	Natural - Earthquake
Soares, J., & Mullings, A. Y. (2009). ‘A we run tings’: Women rebuilding Montserrat. In Women, Gender and Disaster: Global Issues and Initiatives (pp. 250–260). SAGE Publications India Pvt Ltd.	Book Chapter	Qualitative	Latin America and Caribbean	Montserrat	Other	Natural - Volcanic eruption
Solution Exchange - Disaster Management and the Gender Community of Practice (2012). Women and girls: The invisible force of resilience.	Organization Report	Qualitative	South Asia	India	Self-Help Group	Natural - Floods
Story, W. T., Tura, H., Rubin, J., Engidawork, B., Ahmed, A., Jundi, F., Iddosa, T., & Abrha, T. H. (2020). Social capital and disaster preparedness in Oromia, Ethiopia: An evaluation of the “Women Empowered” approach. Social Science & Medicine, 257, 111907.	Peer-reviewed Journal Article	Quantitative	Sub-Saharan Africa	Ethiopia	Other Savings Group	Natural - Drought
Tawodzera, G. (2012). Urban household survival and resilience to food insecurity in crisis conditions: The case of Epworth in Harare, Zimbabwe. Journal of Hunger & Environmental Nutrition, 7(2–3), 293–320.	Peer-reviewed Journal Article	Mixed Methods	Sub-Saharan Africa	Zimbabwe	Other	Economic - Massive inflation
Tol, W. A., Leku, M. R., Lakin, D. P., Carswell, K., Augustinavicius, J., Adaku, A., Au, T. M., Brown, F. L., Bryant, R. A., Garcia-Moreno, C., Musci, R. J., Ventevogel, P., White, R. G., & van Ommeren, M. (2020). Guided self-help to reduce psychological distress in South Sudanese female refugees in Uganda: a cluster randomised trial. The Lancet Global Health, 8(2), e254–e263.	Peer-reviewed Journal Article	Mixed Methods	Sub-Saharan Africa	Uganda	Self-Help Group	Conflict - Conflict
Weingärtner, L., Pichon, F., Simonet, C. (2017). How self-help groups strengthen resilience: A study of Tearfund’s approach to tackling food insecurity in protracted crises in Ethiopia. Overseas Development Institute (ODI) Report.	Organization Report	Mixed methods	Sub-Saharan Africa	Ethiopia	Self-Help Group	Natural - Drought, floods, hailstorms
WHO. (2018). Women join hands to oust ebola from the Democratic Republic of the Congo. WHO | Regional Office for Africa.	Blog	Qualitative	Sub-Saharan Africa	Democratic Republic of the Congo	Unspecified	Health - Ebola
Wineman, A., Mason, N. M., Ochieng, J., & Kirimi, L. (2017). Weather extremes and household welfare in rural Kenya. Food Security, 9(2), 281–300.	Peer-reviewed Journal Article	Quantitative	Sub-Saharan Africa	Kenya	Other Savings Group	Natural - Drought
Yaron, G., Wilson, D., Dumble, S., & Murphy, B. (2017). Measuring changes in household resilience as a result of BRACED activities in Myanmar. Building Resilience and Adaptation to Climate Extremes and Disasters (BRACED). London: UK.	Organization Report	Quantitative	East Asia and Pacific	Myanmar	VSLA; Other Savings Group	Natural - Climate extremes and disasters
Yonder, A., Akcar, S., & Gopalan, P. (2005). Women's participation in disaster relief and recovery. Population Council.	Organization Report	Qualitative	South Asia	India	Multiple	Natural - Earthquake

To complement and deepen our analysis, we provide an in-depth description of how women’s groups responded to the Ebola epidemic in sub-Saharan Africa. We include both the 2014–2016 Ebola Virus Disease (EVD) outbreak in West Africa as well as the 2018–2020 outbreak in the Democratic Republic of the Congo (DRC), which—similar to COVID-19—devastated families, disrupted economic and social activities, and resulted in government policies that placed restrictions on mobility, large gatherings, and livelihoods ( Langlay, 2014; WHO, 2018). This spotlight on the Ebola crisis allows for a more detailed exploration of the interaction between women’s groups and a covariate health shock that requires social distancing, providing in-depth context to complement the findings for each of our research questions.

### Findings

Our included literature, as shown in Table 2, are diverse in geography, type of women’s group, and shock. ^2^ Though women’s groups in sub-Saharan Africa and South Asia are the most widely represented, studies are included from women’s groups in low-and middle-income countries across four continents. The groups in the included studies demonstrate a wide variety of goals and organizing purposes—indeed, many groups formed around multiple livelihoods, health, and financial interests. In terms of the types of shocks represented in the sample, we find more literature on natural disasters (56 out of 90 studies) than other acute covariate shocks ( *e.g.,* 15 studies on health shocks, 10 on conflict shocks, and seven on economic shocks). The diversity in our included literature allows for an examination of the varied interactions between different types of women’s groups, acute covariate shocks, and the resilience of group members and their communities. We also purposively included studies with outcomes in a wide range of domains, including domains that may not be immediately obvious when discussing resilience. For example, we included outcomes related to pregnancy risks for adolescent girls, which can increase without the protection of time in school during pandemics ( Bandiera *et al.,* 2019).

**Table 2.** Overview of included literature.

Documents Coded (90)	Peer-reviewed Journal Article (38) Organization Report (23) Blog/News Article (7) Book Chapter (11) Working Paper/Dissertation/Thesis/Conference Paper (11)		
Geography	Sub-Saharan Africa (37) South Asia (33) East Asia & Pacific (8) Latin America & Caribbean (4) Middle East & North Africa (1) Multiple (7)		
Type of Shock	Natural (56) Health (15) Conflict (10) Economic (7) Multiple (2)		
Type of Women’s Group	Self-Help Groups (27) VSLA & Other Savings Group (26) Microfinance Groups (3) Collective Action & Grassroots Groups (4) ELA and other adolescent girls’ groups (3) Multiple* (14) Unspecified (7) Other (6)		
Organizing Purpose	Financial (31) Health (5) Livelihoods (3) Multiple (43) Other (8)		
Methodology	Quantitative (17) Qualitative (52) Mixed Methods (15) Review (6)		
**Question for document coding**		**Yes**	**No**
Peer Reviewed?		38	52
Discusses the effect of shocks on women's group activities and/or resources?		29	61
If Yes: Positive (1) Negative (25) Neutral (1) Mixed (2)			
Discusses group response or adaptation to shock?		25	65
Discusses women’s groups playing a role in community response?		37	53
Describes the role of women’s groups in the resilience of members to shocks?		46	44
If Yes, list of resilience indicator(s) discussed: Food Security, Consumption Smoothing, & Saving (15) Psychological/Mental State (8) Credit (1) Income, Economic Security, & Employment/Migration (8) Composite Index (2) Pregnancy (2) Disaster Preparedness (1) School Enrollment & Attendance (3) Empowerment/Status (1) Height-to-Age Ratio (1) Access to resources/information (1)			
Methodology includes a comparison group?		20	70
If Yes, list of resilience indicator(s) discussed: Food Security, Consumption Smoothing, & Saving (10) Psychological/Mental State (4) Credit (1) Income, Economic Security, & Employment/Migration (4) Composite Index (2) Pregnancy (2) Disaster Preparedness (1) School Enrollment & Attendance (3) Height-to-Age Ratio (1) Access to Resources/Information (1)			

*Frequently incudes savings, lending, and/or advocacy components

Quantitative and mixed methods research on the impacts of acute covariate shocks to women’s groups—and any resilience that groups may provide to members—is sparse. The majority of the literature available are qualitative in nature, and few studies are peer-reviewed. Out of 90 documents included in our sample, we identified 38 peer-reviewed journal articles, of which 32 used a quantitative or mixed methods research approach.

***How do shocks affect women’s groups?*** This section discusses evidence on the effects of shocks on women’s groups activities and resources, features and adaptations through which women’s groups may increase resilience to shocks, and how shocks may serve as the impetus for new women’s groups. 

*The effects of shocks on women’s group activities and resources, and group resilience*

The evidence on the effects of acute covariate shocks on women’s groups suggests consistently negative impacts on women’s group resources and activities (25 out of 29 documents reporting on this research question indicated an adverse impact on group resources and/or activities), indicating that covariate shocks can adversely affect group resilience. Women’s groups were unable to meet as frequently, ceased certain activities and services, and occasionally dissolved altogether ( Bandiera *et al.,* 2019; BARA & IPA, 2013; Heltberg *et al.,* 2013; Langlay, 2014; Pollard, 2003; Soares & Mullings, 2009). Some women’s groups changed their group function and adapted their activities to support community responses to a covariate shock, such as engaging in emergency warning communications ( Féron, 2020; Mulyasari & Shaw, 2014), information dissemination ( CARE, 2020a), and implementation of rescue and relief programming ( Mehta, 2009) – and this is discussed in greater detail in Section *How Do Women’s Groups Support Community Responses to Shocks?*.

Covariate shocks tend to have deleterious effects on group resources and resilience due to the majority of members experiencing difficulty in contributing to group funds ( *i.e.,* through savings contributions or loan repayments) while simultaneously needing to draw upon group financial services such as credit and social insurance to cope with shocks ( Androsik, 2020; Atela *et al.,* 2018; Bermudez & Matuszeski, 2010; Gash & Gray, 2016; Heltberg *et al.,* 2013; Langlay, 2014; Nannozi, 2019; Nayar & Faisal, 1999). This drastic reduction in the supply of group resources, combined with a sudden increase in demand, may pose a serious challenge to group sustainability and resilience. Covariate shocks may also increase uncertainty around the costs and benefits of group membership. For example, a study during the height of the HIV/AIDS epidemic in South Africa found that individuals were less likely to continue membership in informal risk sharing networks due to the increased likelihood that other group members would die before repaying their loans ( Linkow & Rentschler, 2016). Similarly, a women’s group affected by an acute covariate shock faces a sustainability challenge if members perceive that the cost of group membership is not offset by the anticipated benefits of pooled resources.

*Features and adaptations of women’s groups to increase resilience to shocks*

There is limited evidence specifying the ways in which certain group models or implementation features can contribute to the ability of the group, and/or its members, to withstand shocks, but the existing literature provides some insights into the importance of organizational linkages, flexible policies, and social capital. The primary indicator of a group’s ability to persist and function during a covariate shock is the presence of a formal linkage between the women’s group and NGOs, financial institutions, and/or other women’s groups ( Coppock & Desta, 2013; Demont, 2013; Demont, 2022; Khandker *et al.,* 2015). Linkages to external financial services and resources allow for uninterrupted access to credit for group members, as the resource pool is not constrained to the shock-affected women’s group; that is, the sudden increase in demand for resources during a shock does not threaten group sustainability because the linkage to an external organization prevents a simultaneous decrease in the resource supply.

We also found some evidence that women’s groups adapted their policies around member contributions and share-outs in an effort to mitigate the impact of shocks. Some savings groups timed the share-outs to coincide with the beginning of the lean (pre-harvest) season ( BARA & IPA, 2013; Brunie *et al.,* 2014), while other savings groups implemented shorter loan cycles and new emergency funds to better cope with recurrent shocks ( CARE, 2020a). Women’s groups in India set up a new, banked resilience fund in Odisha after cyclone Fani ( Ghosh, 2019), and the Self Employed Women’s Association (SEWA) created a Livelihood Security Fund tailored to the needs of members living in disaster-prone areas of Gujarat, India ( Benni & Barkataky, 2018). Another adaptation includes the implementation of increased flexibility around contribution amounts during a negative shock, but this adaptation highlights a possible tension between the resilience of groups ( *i.e.,* the group infrastructure and functioning withstanding the shock) and the resilience of group members ( *i.e.,* individual group members withstanding the shock). For example, Demont (2022) found that policies of compulsory savings were central to group resilience after covariate weather shocks, but Khandker, Khalily, and Samad (2015) found that more flexible policies ( *e.g.,* flexibility on loan repayment) were important for individual resilience during shocks.

Descriptions of women’s groups’ adaptation to shocks rarely speak to the implications of these adaptations, such as how these changes may alter the women’s group implementation or effectiveness, or whether these adaptations are sustained after a crisis. A notable exception is Ray-Bennett (2010), who examined how a policy change that prioritized women’s group resources for its most vulnerable members during a shock produced unintended adverse consequences, including group tension and conflict. After a major cyclone hit India in 1999, the NGO managing SHGs in the affected region implemented a policy known as a “vulnerability analysis” approach, which targeted group resources such as credit to poor group members over non-poor members of the same caste. While the intent of this approach was to promote equity, it resulted in the less vulnerable members exhibiting “extreme hostility” towards more vulnerable members that received priority. They “exerted extreme pressure” on the vulnerable recipients to deposit savings and repayments regularly regardless of their difficulties, and, if they failed, were met with "verbal abuse and fiery argument" (p. 252). Ultimately, the most vulnerable were unable to receive any further credit – which "reproduced and re-intensified local gender and class hierarchies, with more privileged women receiving far greater benefit than the poorer" (p. 253). SHGs that did not adopt the vulnerability analysis appeared to fare better than those that did, as this helped to "diffuse the pressure of the least vulnerable group members on the activities of the SHG" (p. 253). The findings of Ray-Bennett (2010) reveal that the implications of women’s group adaptations to shocks is an important—yet understudied—topic in the literature.

Women’s group respondents facing recurrent droughts in Northern Kenya also cited a number of factors important for group sustainability that are closely related to the social capital embedded in groups, including unity and social cohesion, transparency and accountability of group leadership, and the appropriate balance of incentives and discipline in order to “instill an ethos of shared rights and responsibilities” ( Coppock & Desta, 2013, p. 100).

*Shocks as an impetus for the formation of groups*

We also examined how shocks may serve as the impetus for new women’s groups to form. Women’s groups may form organically after a shock as a way for members to provide psychosocial support to one another ( Berry, 2015; Huang & Wong, 2013; Sim *et al.,* 2019; Tol *et al.,* 2020), to pool resources for survival ( Corbin & Hall, 2019; Falk, 2014; Porter, 2001; Tawodzera, 2012), and to advocate for inclusion in community response ( Clissold *et al.,* 2020; Fordham *et al.,* 2011).

In some cases, large covariate shocks such as natural disasters and war may attract NGOs to a region to distribute assistance. For example, BRAC began operations in Bangladesh in 1972 as a small-scale relief and rehabilitation project to help refugees after the Bangladesh Liberation War of 1971 ( Chowdhury & Bhuiya, 2004). Because many NGOs prioritize women in their aid response, an increase in NGO activity may produce an increase in the proliferation and activity of women’s groups ( Kruks-Wisner, 2011; Yonder *et al.,* 2005). Kruks-Wisner describes how the Tamil Nadu state in India experienced “two tsunamis” – the initial Indian Ocean tsunami of December 2004, followed by “a wave [that] pumped unprecedented amounts of aid, materials, and personnel into the affected region” (2011). Many of these newly-arrived NGOs worked with SHGs to provide support and connect women to government officials for advocacy purposes during the recovery process. The relationship between women’s groups and NGOs is bidirectional, as NGOs may establish women’s groups as part of their organizational response, and groups of women may start groups or formalize existing groups after a disaster in order to attract NGO partnerships. For example, after civil conflict in Liberia, many women organized into women’s groups in an “entrepreneurial attempt” to access donor funding that prioritized disbursement to women ( Fuest, 2008).

***How do women’s groups mitigate the effects of shocks for members?*** We next reviewed the literature to assess the extent to which participation in women’s groups is associated with member resilience during an acute covariate shock. We found 46 documents that included evidence on the role of women’s groups in the resilience of members to covariate shocks. Most of this evidence (26 documents) consisted of qualitative interviews with group members and focused on member experiences, without comparison to a control population of non-group members or women without access to groups. These interviews do not provide quantifiable evidence of resilience, but the shared experiences of the women interviewed supply valuable detail and context often lacking in quantitative studies for how group mechanisms produce resilience. For example, women’s groups that formed in the wake of the Rwandan genocide testified to the importance of the group for collective grieving and emotional support as a way to cope with the tragedy of lost family members and displacement ( Berry, 2015). Members of economic SHGs stressed the importance of group membership in accumulating savings and group-based credit to better absorb economic losses due to natural disasters or rainfall shocks – that is, economic SHGs contributed to members’ absorptive capacity ( Coppock & Desta, 2013; Gash & Gray, 2016), and members of agricultural groups attributed their ability to adapt to price shocks ( *i.e.,* adaptive capacity) to shared group storage resources and trainings from group-affiliated NGOs ( Nannozi, 2019).

To address the empirical evidence on the extent to which women’s groups increase resilience to shocks, we limited our analysis to studies with a control or comparison group. Table 3 contains a summary of the included empirical studies (n=20).

**Table 3.** Summary of empirical studies (n=20).

Citation	Country	Women’s Group Intervention	Shock	Identification strategy	Design	Key outcome measures (describe)	Key results	Other results
Bahadur, A., Lovell, E., & Pichon, F. (2016). *Effectiveness in building resilience: Synthesis * *report for Oxfam’s resilience outcome area*. Oxfam Research Reports. Retrieved from https:// oxfamilibrary.openrepository.com/bitstream/ handle/10546/620103/er-effectiveness-resilience- building-080216-en.pdf?sequence=1&isAllowed=y	Senegal (central area)	Farmers received comprehensive intervention, which included savings groups (as well as weather insurance, disaster risk reduction activities, access to credit, and agricultural support). Participants in savings groups were “primarily women.” Impacts applied to the whole intervention; they were not attributable to savings groups alone.	Lean season in arid regions; high levels of climate-related food insecurity. Study conducted 2013–15.	Not specified.	Design not specified. Results given for beneficiary households and comparison households.	Coping Strategy Index (CSI): Measures how households cope with food shortages. Higher score means more frequent/intense coping mechanisms. Food Consumption Score (FCS): Reflects number of meals per day; higher percentage means more food consumption.	CSI: Both groups increased their strategies for coping with food insecurity challenges. Beneficiary households increased CSI scores by 1.7 percentage points, while comparison households increased CSI scores by 7.8 percentage points (no indication of statistical significance provided). Project households were more likely to use savings as a coping strategy (instead of borrowing, using credit, *etc.*). FCS: Both groups decreased food consumption, on average. Comparison households decreased FCS from 56% to 29% from 2013 to 2015, while project households decreased FCS from 59% to 54% (no indication of statistical significance provided).	Beneficiary households were more likely to use solar energy and to produce higher rice yields than comparison households, but it is unclear whether these outcomes were due to savings group participation.
Bandiera, O., Buehren, N., Goldstein, M. P., Rasul, I., & Smurra, A. (2019). *The economic lives of * *young women in the time of Ebola: Lessons from* * an empowerment program*. The World Bank. Retrieved from https://elibrary.worldbank.org/doi/ pdf/10.1596/1813-9450-8760	Sierra Leone (Port Loko, Kambia, Moyamba, and Pujehun)	BRACs Empowerment and Livelihood for Adolescents (ELA). Club for girls (ages 18–25) to meet and gain livelihood skills, trainings, and reproductive health knowledge.	2014 Ebola outbreak. Villages were categorized as high or low Ebola- disruption villages (binary shock indicator). Index score of disruption includes school and health facility closures or disruptions; villages with index score above 75th percentile scored as “high” disruption (17% of villages in sample).	2x2 factorial design—randomized controlled trial (RCT) for treatment, quasi- random for shock.	ELA randomly assigned to 150 villages; 50 control villages. Baseline data collected in early 2014 (start of outbreak); endline in 2016 (near end of outbreak). Young cohort (ages 12–17) and older cohort (ages 18–25). *N* = 4,790 (17% attrition; no differential attrition of treatment group). Difference-in-differences (DD) analysis, interaction term for treatment*shock estimate.	School enrollment: whether or not a respondent was enrolled in school. Literacy skills: self-reported abilities to read simple text; reading comprehension; writing complete sentences; etc. Aggregated and scaled on 0–100 index (average baseline score 24.6). Numeracy skills: self-reported abilities to perform basic counting; simple calculations; working with fractions; etc. Aggregated and scaled on 0–100 index (average baseline score). Out-of-wedlock pregnancy: from survey.	ELA mitigated the impacts of Ebola disruption. For the younger cohort, school enrollment dropped 16.6 percentage points in highly disrupted control villages but declined only 8.1 percentage points in disrupted-treatment villages ( *p* < 0.1). For younger girls, Ebola disruption reduced literacy (numeracy) scores by 12.1 percentage points (7.3%) in control villages, but ELA offset around 73% (99%) of these reductions ( *p* < 0.01). For the older cohort, 93% of the loss in numeracy skills was offset by ELA ( *p* < 0.01). Out-of-wedlock pregnancies rose by 7.2 percentage points in high-disruption villages, but this increase was completely mitigated in treatment villages ( *p* < 0.05).	The older cohort realized a significant increase in transactional sex and rape in high-disruption treatment villages (5.4 percentage points), but there was no corresponding increase in pregnancy, which suggests that the treatment increased contraceptive knowledge and use.
Bandiera, O., Buehren, N., Goldstein, M. P., Rasul, I., & Smurra, A. (2020). *Do school closures during* * an epidemic have persistent effects? Evidence* * from Sierra Leone in the time of Ebola*. Retrieved from http://www.homepages.ucl.ac.uk/~uctpimr/ research/ELA_SL.pdf **(Follow-up to Bandiera *et al.* [2019])**	Sierra Leone (Port Loko, Kambia, Moyamba, and Pujehun)	BRACs ELA. Club for girls (ages 18–25) to meet and gain livelihood skills, trainings, and reproductive health knowledge.	Same as Bandiera *et al.* (2019) but renames “high disruption” village to “high pregnancy risk” village.	2x2 factorial design— RCT for treatment; quasi-random for shock (DD analysis).	See info for Bandiera *et al.* (2019). Included additional round of data collection in 2019–20 of 2852 respondents (~ 60% of original sample; no differential attrition of treatment group). Also included surveys of 1368 partners of original sample.	Included follow-up data for school enrollment, contraceptive use, and pregnancy outcomes from Bandiera *et al.* (2019). Included additional partner characteristics (aversion to gender-based violence [GBV], education, *etc.*).	Short-term results are not substantively different from those reported in Bandiera *et al.* (2019). At follow-up: Highly disrupted villages had persistently lower enrollment rates and higher pregnancy rates than villages less impacted by Ebola. The ELA clubs also had positive effects on education after Ebola with a more limited fall in school enrollment post-pandemic in treatment villages than in control villages. For the older cohort, the positive treatment effect on increased contraceptive use in disrupted villages remained statistically significant.	The survey of partners revealed more favorable traits among partners of treatment group women compared to control group women, but this analysis was not interacted with the disruption of the Ebola shock.
Bureau of Applied Research in Anthropology & Innovations for Poverty Action. (2013). *Final impact* * evaluation of the saving for change program in* * Mali, 2009–2012*. Retrieved from https://www. freedomfromhunger.org/sites/default/files/ SavingforChangeMaliResearchFullReportMay2013. pdf	Mali (Ségou)	Women-only groups, self- managed savings and credit groups (Saving for Change program). No external capital. Share-out timing often coincided with periods of high cash-flow requirements.	Lean period (beginning in May/June) is the only covariate shock that was quantitatively analyzed. Study period was 2009–12.	RCT.	500 villages randomly assigned to treatment or control. *N*=5954; households; included big households of multiple subfamily units and small households with one head of household. Baseline and endline surveys administered to full sample. Shock-specific analysis was conducted with high-frequency sample: 600 randomly selected women from both treatment and control groups. High-frequency sample was surveyed every two weeks or every three months, depending on survey group.	Food consumption: Measured with one-week recall survey. Administered to high-frequency subsample. Frequency for this specific survey component is unclear: somewhere between every two weeks and every three months between June 2010 and January 2012. Food security: Adapted for high-frequency sample from 17-question Freedom From Hunger food security index. Three-month recall. Frequency for this specific survey component is unclear: somewhere between every two weeks and every three months between June 2010 and January 2012.	Food consumption: For the high-frequency sample, households in treatment villages experienced a smaller decline in food consumption during the lean season. This effect is statistically significant only for the subset of small households (not full sample or large households). That is, small households in treatment villages consumed, on average, 0.39 USD more than small households in control villages ( *p* < 0.05). *Note*. Unclear unit/transformation for coefficient 0.39 USD.	No significant differences in food security index for treatment households during the lean season.
Bass, J., Murray, S., Cole, G., Bolton, P., Poulton, C., Robinette, K., ... Annan, J. (2016). Economic, social and mental health impacts of an economic intervention for female sexual violence survivors in Eastern Democratic Republic of Congo. *Global* * Mental Health, 3*.	DRC (South Kivu)	Women-only village savings and loan associations (VSLAs); received training but no outside capital from nongovernmental organizations (NGOs).	Civil conflict (ongoing since 2004; study conducted in 2010). Conducted specifically among victims of sexual violence (SV) in conflict-affected communities.	RCT.	Study-eligible women (SV survivors; *n*=301) participated in VSLAs alongside other women from community. VSLAs were randomized to immediate start (treatment) and wait-list controls. Outcomes measured with baseline/endline questionnaire. 17% attrition (per protocol analysis conducted).	Per capita food consumption: measured with seven-day recall survey, including food purchases and food produced by household. Internalized stigma: measured with survey; produced summary score of 0–3.	Per capita food consumption: 25% greater increase ( *p*=0.01) from baseline to endline for treatment group compared to controls (but control group still had higher expenditure in CDF at both time periods; highest consumption per capita is still < 1 USD). Internalized stigma: Both groups realized reductions in stigma, but women in treatment group reported more than 10% greater reduction in internalized stigma than those in control groups ( *p*=0.038).	Marginally significant results include women in treatment group having about 1.5 more animals for breeding and a smaller reduction (17%) in paid hours worked than women in control group. Nonsignificant outcomes: Mental health functioning and additional social outcomes.
Buehren, N., Chakravarty, S., Goldstein, M., Slavchevska, V., & Sulaiman, M. (2017). *Adolescent* * girls’ empowerment in conflict-affected settings:* * Experimental evidence from South Sudan*. CSAE Conference Paper	South Sudan	Adolescent girls—BRAC Adolescent Girls Initiatives (AGI). Groups for socializing, livelihoods, and life skills training.	Conflict of December 2013 (affected about half the sample). Girls were affected if they answered “yes” to at least one of seven questions (house looted, household member died, etc.).	RCT.	120 eligible villages were randomized to treatment or control. Cluster random sample of 35 girls from each village was surveyed. *N* = 3219 baseline respondents (after dropping one area due to security concerns). Baseline survey in 2010. Extensive attrition resulted in random cross- section endline in 2014–15 (not same girls as baseline). *N* = 2273 endline respondents; *n* = 1558 who answered all relevant survey questions. Intention-to-treat (ITT) impact estimated using endline cross-section only; model included interaction conflict*treat. Model tested program impact for 40 outcomes; no correction for multiple hypothesis testing.	Employment: Several subcomponents, including any income-generating activities (IGAs), self-employment, wage employment, farm/non-farm self-employment, income, hours worked, control over earnings. School enrollment: binary variable (currently enrolled in school=1).	Employment: Beneficial effects of program on employment were wiped out for girls affected by the conflict. School enrollment: Conflict led to 6.8 percentage-point decrease in control villages but no significant decrease in enrollment in treatment villages ( *p* < 0.1).	Many other outcomes examined (human capital, food security, assets, empowerment, savings, gender roles, *etc.*). No meaningful differences between treatment and control groups for conflict- affected girls.
Christian, P., Kandpal, E., Palaniswamy, N., & Rao, V. (2019). Safety nets and natural disaster mitigation: evidence from cyclone Phailin in Odisha. *Climatic * *Change, 153*(1–2), 141–164. Retrieved from https:// doi.org/10.1007/s10584-018-02364-8	India (Odisha)	Women-only SHGs (formed by Targeted Rural Initiatives for Poverty Termination and Infrastructure and the National Rural Livelihoods Mission); SHGs were federated and linked to external public/private services.	Cyclone Phailin (October 2013). Rainfall shock measured continuously as natural log of deviations from monthly historical median rainfall (validated with use of surveys about flooding, *etc.* during Phailin).	Quasi-experimental (non-random assignment). Triple difference model to estimate ITT effect.	Assigned to treatment versus comparison at village/community level. Baseline balance table suggests comparability. Baseline survey in 2011 and endline survey in 2014 ( *N* = 2874 households). 20 outcomes examined; no correction for multiple hypothesis testing.	Food consumption: Followed the Indian National Sample Survey. Non-food consumption: Followed the Indian National Sample Survey.	Food consumption: No significant difference between treatment and comparison households similarly impacted by shock; group membership did not mitigate reductions in food expenditure after Phailin. Non-food consumption: Households in treatment villages spent, on average, about 785 rupees more per capita than comparison households ( *p* < 0.05). That is, group membership offset the decline in non-food expenditure after Phailin.	Large but nonsignificant coefficient on expenditures for women’s goods suggests that group membership may “buffer” some of the reduction in this category. No significant differences in other categories of expenditures. Significant increase in number of current loans held by treatment households. Of six civic engagement outcomes, one was significant: Women in treatment villages were more likely to be aware of the last village council meeting.
De, S. (2011). *The whims of Indian monsoons: * *Long-term health consequences of early childhood* * exposure to the Indian drought of 2002*. Young Lives Student Paper.	India (Andhra Pradesh)	Targeted program (IKP) to extend SHGs to rural poor women (below the poverty line). Program is government- sponsored, and SHGs are bank linked.	Drought of 2002 (driest monsoon season since 1960). Districts were scored as having been affected by the drought if rainfall was at least 20% deficient from long period average (shock is binary variable).	Longitudinal analysis; non-random assignment to treatment. Panel data (three waves) analyzed with differenced Gaussian mixture model (GMM) model.	Six (poorest) of 22 districts were enrolled in program during drought (not random). These six districts composed treatment group; other 16 districts served as comparison group. Three waves of Young Lives survey (2002; 2007; 2009–10) collected anthropometric data on children. Balanced panel: Young cohort (born 2001–02): *N*=1259. Older cohort (born 1994–95): *N*=802. Approximately 30% attrition (non-random attrition with no correction, but author sees no evidence of attrition bias on outcome data). Differenced GMM model with twice-lagged health status to estimate impact of household having access to IKP program during drought.	Health status: World Health Organization height-for-age (HAZ) z score.	Health status: The drought had significant negative impacts on both treatment and comparison groups (more severe for younger cohort). The coefficient on the interaction term of drought * program is positive but not significant.	
Demont, T. (2013). *Poverty, access to credit and* * absorption of weather shocks: Evidence from Indian* * self-help groups*. CRED Working Paper. Demont, T. (2022). *Coping with shocks: How self-help* * groups impact food security and migration. World* * Development, 155, 105892.*	India (Jharkhand)	Women-only SHGs; bank linked	Rainfall shock (2004–09). Measured as a continuous variable; standardized difference from norm. A 1 standard deviation (SD) reduction in rainfall (drought) resulted in an average loss of over 50% of agricultural yields. Rainfall shock (2004–09). Measured as a binary variable. Shock is present if rainfall was at least 0.5 SDs below norm (contrast with Demont, 2013).	Quasi-experimental. Random allocation at village level to treatment and control. Random selection of households analyzed using DD model. Self-selection into SHGs in treatment villages.	Study participants were randomly selected from within a stratified random sample of villages. A random sample of households from comparison villages (no SHGs) was also included. Total sample size was 1080 households. Three rounds of household panel data analyzed through DD technique. Same sample as Demont (2013). In the 2022 study, treatment is defined as a household within a treatment village instead of a household participating in an SHG (contrast with Demont, 2013).	Food consumption: Measured with one-week recall survey, administered right after harvest (when households were likely to have extra) Food security: Annual survey asks household to recall each month of past year: In month X, could all household members enjoy three meals per day? Credit: Survey about loans taken in prior two years.	After years with a negative rainfall shock, households in comparison villages lost, on average, 1.6 months of adequate food, while households in treatment villages lost 0.9 months on average. That is, households in treatment villages realized a 59% reduction ( *p* < 0.05) in loss of food security during a rainfall shock. In the 2022 study, households in treatment villages experienced an average of one month without sufficient food, compared to two months in control villages (p < 0.10). After a shock, households in treatment villages increased their probability of borrowing by ~16%, while control households decreased their probability of borrowing by ~50% - indicating that treatment villages had better access to credit after a shock.	Households in treatment villages were 35% more likely to migrate for work (and realized over a 40% increase in migration income or remittances) in the year after a rainfall shock than households in comparison villages. In the 2022 study, these estimates increase to 50% more likely to migrate, producing a 60% increase in income.
Garikipati, S. (2008). The impact of lending to women on household vulnerability and women’s empowerment: Evidence from India. *World * *Development, 36*(12), 2620–2642. Retrieved from https://doi.org/10.1016/j.worlddev.2007.11.008	India (Andhra Pradesh)	Women-only SHGs; bank linked	Drought (2001–02). Respondents were asked about their vulnerability during “the last drought.” Drought as a shock is not measured quantitatively; it is perceived by the respondent.	Not specified.	Study design not specified. Surveyed 117 households (participated in SHG) and 174 comparison households in 2001; surveyed again in 2002. Sample included only married-couple households, and male/female respondents were chosen at random. Surveys were used to construct binary vulnerability and empowerment indicators. T-tests determined significant differences in means.	Drought-related vulnerability: self-perceived ability to meet needs. Points were given for each type of need the household was able to meet during the last drought ( *e.g.,* all food needs, health needs), as well as if the household avoided selling assets and expected to cope similarly in next drought. Instrument was then collapsed into binary indicator for vulnerable/not vulnerable, with cutoff around lowest 30th percentile.	T-test revealed significant difference in means of drought-related vulnerability between treatment and comparison households. 40% of treatment households were not vulnerable compared to 26% of comparison households ( *p* < 0.05). Logit regression shows that duration in program is associated with higher probability of no vulnerability.	Treatment households were more likely to have livelihood diversification. Participation in SHGs is also associated with lower levels of women’s empowerment, sometimes referred to as the “impact paradox”.
Jahns, E. (2014). *Savings groups, shocks and coping* * strategies: The case of poor rural households in El* * Salvador* (Doctoral dissertation, Fletcher School of Law and Diplomacy, Tufts University).	El Salvador (rural eastern; communities with high poverty rates)	Savings groups facilitated by NGO as part of larger intervention involving agricultural training and resources. Most groups consisted only of women, some had only men, and some were mixed. Some groups also incorporated emergency funds and/or loans. No groups received outside capital.	High global market prices and bad harvests in 2010 led to the Hungry Season of 2011.	Non-random assignment to treatment; non- random selection into study. Comparison communities chosen for matching characteristics. Linear probability model used to estimate association of treatment community with outcome.	Case study with mixed-methods nested design. 13 communities selected for maximum variation (eight with savings groups; five comparison selected on matching characteristics). Heads of households from each community randomly selected for study. Qualitative interviews conducted in 2010–11 to structure quantitative survey instrument administered in 2012. Linear probability model used to estimate association between living in community with savings groups and successful coping strategies. *N*=276 households.	Successful coping: Binary variable. A household coped successfully with the hungry season of 2011 (April–August) if no one in the household experienced hunger in the previous 12 months (surveyed in 2012).	The coefficient on savings groups was consistently positive: Households in communities with savings groups were 6.5 to 9.5 percentage points more likely to cope successfully with the shock (depending on choice of model). However, significance of the coefficient is marginal at best and varies with choice of model covariates.	Poorer households were 2.7 percentage points more likely to have successful coping strategies (marginally significant; not robust for model with community-level controls). Household heads with at least one year of schooling had a 9.9 percentage-point increase in probability of having successful coping strategies ( *p*=0.045).
Kaboski, J. P., & Townsend, R. M. (2005). Policies and impact: An analysis of village-level microfinance institutions. *Journal of the European Economic * *Association, 3*(1), 1–50. Retrieved from https://doi. org/10.1162/1542476053295331	Thailand (rural and semi-urban)	Microfinance institutions (multiple types). Production credit groups (PCGs) are less likely to contain the poorest in a village but more likely to consist of mostly women. PCGs operate like VSLAs; may receive start-up capital but are not linked to larger intermediation network. Women’s groups overlap in saving/lending functions with PCGs but usually also contain a training/livelihoods element for example with a focus on rice banks and buffalo banks.	A “bad year.” Year is identified through household self-report— lowest income year in last five years (1992–97). Unclear whether this represents covariate shock.	Non-random assignment to treatment. Cross- sectional survey of random selection of households. Two- stage least squares (2SLS) and maximum likelihood estimation (MLE) models used to estimate association of microfinance institution with outcome.	Cluster random selection of 192 survey villages, 15 households from each village ( *n*=2880). Household survey administered in May 1997 (cross-sectional). 2SLS and MLE models constructed to measure association of microfinance institution with likelihood of reducing consumption. Multiple variations of institutions tested; no correction for multiple hypothesis testing.	Reduced consumption/input use: Households were asked to identify the worst income year in the past 5 years and indicate whether they had to reduce consumption or inputs for that year.	Households in villages with microfinance institutions that offered savings services were 12 to 18 percentage points less likely to reduce consumption in a bad year (though significance was not robust to specific savings service evaluated). Analyses focusing only on women’s groups or PCGs (instead of lumping all microfinance institutions together as the “treatment”) did not produce significant differences with regard to consumption smoothing in a bad year.	Households in villages with microfinance institutions that offered emergency services were 20 percentage points less likely to reduce consumption in a bad year.
Karlan, D., Savonitto, B., Thuysbaert, B., & Udry, C. (2017). Impact of savings groups on the lives of the poor. *Proceedings of the National Academy* * of Sciences, 114*(12), 3079–3084. Retrieved from https://doi.org/10.1073/pnas.1611520114	Ghana, Malawi, Uganda (pooled RCTs)	Mostly women VSLAs established by NGOs. Not linked to outside capital.	Drought (2009–11). Annual rainfall less than 1 SD below average for 12 months before endline survey. Only a subset of villages experienced the shock (none in Ghana).	Cluster RCT.	Cluster RCTs (pooled across three countries). Stratified random assignment of 561 village clusters to treatment or control. Households randomly selected ( *N*=15,221). Baseline and endline surveys, studies conducted over a period of 22 to 30 months. Pooled model with ITT estimates, adjusted for multiple hypothesis testing.	Food security: Index composed of five binary indicators, 12-month recall (adult/child reducing food intake adult/ child going a full day without food, borrowing food). Income: Self-reported revenues minus expenses for all IGAs carried out by the household in the 12 months before the survey.	No significant impacts when adjusting for multiple hypothesis testing. Food security: Drought significantly reduced food security for control households by 0.119 SD (adjusted *p* < 0.05), but there was no significant difference between treatment and control households (adjusted *p*=0.26). Income: After the drought, treatment households had, on average, 26.40 USD more in income than control households, but statistical significance disappears when adjusting for multiple hypothesis testing ( *p* < 0.1; adjusted *p*=0.26).	No significant differences between treatment and control groups for business outcomes, asset index, per capita consumption, or community participation index. Women’s empowerment: For households not experiencing a shock, the treatment group displayed increases in women’s empowerment. However, for households experiencing drought, the coefficient on women’s empowerment was negative for treatment households ( 0.119 SD; *p* < 0.05; adjusted *p*=0.26).
Ksoll, C., Lilleør, H. B., Lønborg, J. H., & Rasmussen, O. D. (2016). Impact of village savings and loan associations: Evidence from a cluster randomized trial. *Journal of Development Economics, 120*, 70–85.	Malawi (northern rural)	Gender of groups not specified. VSLAs, no access to outside capital. Article stresses that common VSLA policy is to time the share-out to periods in which households are likely the most resource constrained.	This paper does not focus on a shock, but one outcome of interest is the length of the “hungry period,” a lean season in which household members eat fewer than three meals per day. Data were measured in 2009 and 2011; no discussion of how the hungry period during this time frame compares to average.	Cluster RCT.	Cluster RCT. 46 villages randomly assigned (within strata) to either treatment (NGO-implemented VSLAs) or wait-list control. Baseline and endline household surveys ( *n*=1775 households). 45% of treatment households participated in VSLA; 21% of control households participated in VSLA (spillover). 3% attrition. Outcomes of interest were assessed with four different ITT model specifications (mean difference, lagged, DD, first- difference). *P* values corrected for multiple hypothesis testing. Sample was balanced at baseline.	Length of hungry period: How many months in the past year did household members eat fewer than three meals per day?	There was no significant impact on the length of the hunger period.	The paper examined the impact of VSLA participation on a number of different outcomes, but none of the other outcomes was associated with a shock.
Story, W. T., Tura, H., Rubin, J., Engidawork, B., Ahmed, A., Jundi, F., . . . Abrha, T. H. (2020). Social capital and disaster preparedness in Oromia, Ethiopia: An evaluation of the “Women Empowered” approach. *Social Science & Medicine, 257*, 111907. Retrieved from https://doi.org/10.1016/ j.socscimed.2018.08.027	Ethiopia (Oromia)	Project Concern International’s Women Empowered approach. Women-led VSL; included programming on empowerment and business skills. Does not appear to have included linkages to external capital.	Residents had been affected by 2015–16 drought (one of the worst droughts on record). Study conducted in 2017. Exposure to past shock measured as binary indicator of any loss of income in last three years due to a disaster (> 90% respondents exposed). Also included binary indicator if whole household migrated in search of pasture/water.	Quasi-experimental (self-selection into treatment). Cluster random selection of sample. T tests and Poisson regression used to compare outcomes between groups.	Study district contained eight treatment villages and 19 comparison villages (not randomly assigned). Random selection of 29 women’s empowerment groups within eight treatment villages, followed by random selection of 10 to 11 women from each group (self-selection into groups). In comparison villages, random selection of 10 villages and 28–30 women from each village. *N* = 589. Survey administered in July 2017. T-tests used to compare treatment and comparison for preparedness outcomes. Poisson regression used to control for shock exposure (and other covariates).	Whether or not household had taken actions to prepare for a disaster: single survey question; actions included diversification, insurance, savings, *etc.* Binary variable. Self-perceived preparedness: Survey asked, “How prepared would you say you are for a major natural or man-made disaster in your community?” Those who reported “not prepared at all” were coded as 0; all other levels of preparedness were coded as 1.	Preparedness actions: In a model with no covariates, preparedness actions were 37% more prevalent among women in the treatment group ( *p* < 0.01). However, this finding was not significant in models controlling for social capital and respondent characteristics. Self-perceived preparedness: In a model with no covariates, self-perceived preparedness was 52% more prevalent among women in the treatment group ( *p* < 0.01). However, this finding was not significant in models controlling for social capital and respondent characteristics.	The study also examined the mediating role of social capital in disaster preparedness. Emotional support may have been an important mediator in the relationship between group membership and perceived preparedness.
Tol, W. A., Leku, M. R., Lakin, D. P., Carswell, K., Augustinavicius, J., Adaku, A., . . . van Ommeren, M. (2020). Guided self-help to reduce psychological distress in South Sudanese female refugees in Uganda: A cluster randomised trial. *The Lancet * *Global Health, 8*(2), e254–e263. Retrieved from https://doi.org/10.1016/s2214-109x(19)30504-2	Uganda (refugee settlement in North)	Refugee women (South Sudanese) with at least moderate psychological distress (many exposed to high levels of GBV). Women assigned to groups of 20–30 to receive Self Help Plus intervention. Facilitator- guided intervention was delivered as a group workshop but also contained individual components (meeting with community health worker). Intervention consisted of five weekly workshop sessions.	Conflict in South Sudan (ongoing; study conducted in 2017). Female refugee population with high levels of post-traumatic stress disorder from exposure to GBV and conflict.	Cluster RCT.	Fourteen villages randomly assigned to either treatment or control; 40–60 households randomly selected within each village. *N*=694 women. Participants were surveyed at baseline, immediately after five-week intervention, and at three- month follow-up. 10% attrition (not differential; listwise deletion from analysis).	Psychological distress: Kessler six-item scale. Scores ranged from 0–24. 5 is cutoff for moderate distress, 13 for severe.	Psychological distress: K6 scores were lower, on average, for both treatment and control groups over time. At week 6 (immediately after intervention), women in treatment group scored, on average, 3.25 percentage points lower on the K6 than women in control group ( *p* < 0.001). At three-month follow-up, women in treatment group scored, on average, 1.20 percentage points lower than women in control group ( *p* = 0.04). Subanalysis of women with severe distress (scores of at least 13 on K6) showed significant reduction in proportion of treatment group scoring in the “severe” category at week six and at three-month follow-up. Due largely due to fewer women in treatment group deteriorating than in the control group.	At three-month follow- up, treatment was also associated with improved outcomes on post-traumatic stress and depression symptoms, explosive anger, and functional well-being.
Weingärtner, L., Pichon, F., & Simonet, C. (2017). *How self-help groups strengthen resilience: A * *study of Tearfund’s approach to tackling food * *insecurity in protracted crises in Ethiopia*. Overseas Development Institute (ODI) Report. Retrieved from https://www.odi.org/sites/odi.org.uk/files/resource- documents/11625.pdf	Ethiopia (Ofa and Kindo Koysha)	NGO established SHGs, broad range of activities that included savings and loans. No start-up capital; linkages to external capital not apparent for this study site. Marginalized women were specific target, but groups also included men.	Residents had been affected by 2015–16 drought (one of the worst droughts on record). Study conducted in 2017.	Non-random assignment to treatment; non-random selection of sample. Cross-sectional, qualitative study.	Non-random selection of study participants (SHG members and non-members) from nine study villages ( *n*=252). Qualitative interviews and surveys conducted in 2017 (cross-sectional).	Themes of qualitative interviews included reliance on predatory lenders, risk diversification, and shock resilience.	No quantitative results. Interviews revealed that SHGs provided a way to avoid predatory lenders and that SHG members were better off than non-members in a drought. Diversification in food-supply structures seemed to contribute to higher food security for SHG members. SHG members were also more likely to store foodstuffs or accumulate savings.	SHG members appeared to be more prepared than non-members for future shocks. SHG ability to combat covariate shock was limited because communal resources were strained; draining communal resources during a drought meant less for productive use later. SHG membership is not a replacement for formal social protection during a shock.
Wineman, A., Mason, N. M., Ochieng, J., & Kirimi, L. (2017). Weather extremes and household welfare in rural Kenya. *Food Security, 9*(2), 281–300.	Kenya (southern rural)	Gender not specified. Membership in savings group was coded as 1 if any member of the household belonged to a savings group.	Rainfall, temperature, and wind shocks from 2000–07. Measured as cumulative days over/under thresholds ( *e.g.,* cumulative wind speed days over 5 m/s). Low rainfall was found to be more consistently severe than higher rainfall.	Non-random assignment to savings group. Longitudinal panel regression, with household fixed effects.	Longitudinal household panel survey (2000, 2004, 2007) alongside weather data. Household outcomes were examined, with participation in a savings group examined as a mitigating factor in rainfall deficits (no random assignment). *N*=1264 households. *Note*. Study design was to measure effect of weather shock on household; not designed to identify impact attributable to savings group.	Household poverty status: binary variable indicating whether household was below the poverty line for rural Kenya (income/AE/day ≤ 67 Kenyan Shilling) Household income: income per adult equivalent (AE) per day, measured in Kenyan shillings (Ksh).	Membership in a savings group had a positive but nonsignificant effect on whether or not a rainfall deficit pushed a household below the poverty line (-0.07; *p*=0.16). Membership in a savings group was associated with significantly higher household income during a rainfall deficit (coefficients not reported).	Access to credit was also a significant mitigating factor, but paper does not specify group-based informal credit.
Yaron, G., Wilson, D., Dumble, S., & Murphy, B. (2017). Measuring changes in household resilience as a result of BRACED activities in Myanmar. London, UK: Building Resilience and Adaptation to Climate Extremes and Disasters (BRACED). Retrieved from https://www.itad.com/wp-content/uploads/2018/05/ DFID-BRACED_EA3-Impact-Evaluation_Myanmar_ Final_Shared.pdf	Myanmar	BRACED implemented Community Resilience Assessments (CRAs) that included VSLAs, trainings, and infrastructure. Impacts were estimated for entire intervention and not attributable to VSLAs alone.	Study set in multihazard context. Impact of past climate shocks was still felt, and future shocks are anticipated to grow worse. Most households reported exposure to shock in last 10 years at baseline; < 20% households reported exposure to shock between baseline and endline.	Non-random assignment to treatment. DD model to estimate impact of treatment on outcome.	Stratified random sample of households in treatment and comparison communities. Comparison communities selected based on geographic proximity and criteria similar to those of treatment areas. Baseline (2015) and endline (2017) household surveys. *N*=2168, 7% attrition (not different for treatment or comparison; slightly different for outcome). Impact on outcome (composite resilience index) estimated using DD analysis.	Resilience index: Composite index of five resilience dimensions, measured with 30 questions on household survey. Produced continuous score between 0–1.	Households in treatment villages realized, on average, an 18% increase in overall resilience index scores, while households in comparison villages realized a 14% increase ( *p*=0.002). In a subsample ( *n*=400) of individual intervention components, VSLAs showed significant impact on improved resilience scores in two of eight villages (nonsignificant results in six of eight).	Female-headed households and households with more assets tended to benefit more from intervention (this was not measured specifically with respect to experiencing a shock). No indication that intervention increased food security.

The majority of the empirical studies (13 of 20) examined resilience to a weather shock. These weather shocks varied in terms of severity and acuteness, and this review includes evidence of groups providing resilience to seasonal hunger periods as well as to droughts, monsoons, and other climate disasters.

Resilience was most commonly measured using some indicator of consumption, such as changes in food security or household expenditures, and seven studies provided evidence that women’s group member households tended to be better able to smooth consumption during covariate weather shocks than non-member households ( Bahadur *et al.,* 2016; BARA & IPA, 2013; Christian *et al.,* 2019; Demont, 2013; Demont, 2022; Garikipati, 2008; Karlan *et al.,* 2017). It is important to note that, while consumption tended to be higher for member households than non-member households, membership in a women’s group seldom fully mitigated the effect of the weather shock. That is, the shock reduced consumption for all households, but member households experienced less of a reduction in consumption than non-member households; their increased absorptive capacity provided the ability to absorb more of the shock before reducing consumption. For example, Demont (2022) surveyed households in India about the number of months in the past year that households had to reduce food consumption, finding that households in villages with women’s groups realized nearly 50% less of a reduction in food security during a rainfall shock (compared to households in villages without women’s groups). Christian *et al.* (2019) found no significant impact of women’s groups on food consumption in the year after Cyclone Phailin – but did find that households in treatment villages spent significantly more per capita (785 Rs) on non-food consumption after the shock. Women’s group members also tended to have higher household income after weather shocks ( Demont, 2022; Karlan *et al.,* 2017; Wineman *et al.,* 2017), as well as better disaster preparedness strategies than non-members – indicative of increased adaptive capacity ( Story *et al.,* 2020; Weingärtner *et al.,* 2017; Yaron *et al.,* 2018).

However, two studies using food security and nutrition-related outcomes found no evidence that membership in a women’s group provided benefits during a weather shock. One study examined the length of time households subsisted on less than three meals per day and found no difference between the treatment and control group ( Ksoll *et al.,* 2016). The other study found no significant benefit of group membership amongst households when examining child development outcomes several years post-drought ( De, 2011).

Three studies in sub-Saharan Africa provide mixed results on the ability of group membership to increase resilience to conflict shocks. Victims of conflict-related sexual violence participating in VSLAs in the DRC realized group benefits of increased food expenditure and decreased internalized stigma, but the study found no statistically significant differences between group members and non-group members for additional mental health and economic outcomes ( Bass *et al.,* 2016). Similarly, adolescent girls living in the midst of conflict in South Sudan did not achieve the economic outcomes the group was designed to provide, but they did enroll in school at higher rates post-conflict than non-group adolescents ( Buehren *et al.,* 2017). Neither of these two studies focused on groups specifically designed to mitigate the effects of these conflict shocks, but a third study conducted after civil unrest in Uganda measured the effect of interventions with specific mental health programming by newly formed groups and found that women in these group-based interventions had less psychological distress and better mental health than women in the comparison group ( Tol *et al.*, 2020).

This review also includes two studies on the importance of access to savings and credit through women’s groups during price shocks, as well as a study of adolescent girls’ groups during the Ebola outbreak (detailed in Section Spotlight on Women’s Groups and Ebola). During a year of high price volatility, members of savings groups in El Salvador were 9.5 percentage points more likely to avoid experiencing hunger than non-member households ( Jahns, 2014). Likewise in Thailand, households in villages with microfinance groups were better able to smooth consumption than control villages in periods of widespread economic hardship ( Kaboski & Townsend, 2005).

During shocks to income and resources, access to group-based credit is an important mechanism for a household’s absorptive capacity, as credit can help to smooth consumption, reduce asset loss, and allows borrowers to avoid predatory lenders ( Weingärtner *et al.,* 2017), especially when credit conditions are flexible ( Khandker *et al.,* 2015). However, as described in the previous section, covariate shocks may deplete group resources to the extent that loans are not available to members – especially if the group is not formally linked to a broader network or a financial institution ( Demont, 2013; Gash & Gray, 2016). In cases where group-based credit is not available during a covariate shock, the savings and information sharing mechanisms of women’s groups may be paramount ( Karlan *et al.,* 2017). The accountability and regular commitment of savings groups ensure that members have greater absorptive capacity through accumulated savings, while access to information networks about crop diversification strategies and labor opportunities allows members to increase their adaptive capacity by smoothing income during shocks ( Demont, 2013; Demont, 2022; Karlan *et al.,* 2017). Mechanisms of shifting gender norms, empowerment, and collective action and mobilization may contribute to the transformative resilience of women’s group members during shocks ( Gram *et al.,* 2019), which is described in more detail in Section *How Do Women’s Groups Support Community Responses to Shocks?*.

However, the literature also revealed certain limitations with regard to the ability of women’s groups to contribute to individual resilience during a shock. Women’s groups may not benefit all members equally, and improved resilience for member households does not always equate to improved resilience for the woman herself. There are very few subgroup analyses in the reviewed studies, and we know little about the potential differential impacts of women’s groups on members. Compulsory savings may also reduce the ability of the poorest women to join groups even when this mechanism contributes to increased member resilience. Additionally, Garikipati (2008) and Karlan *et al.* (2017) find that group-attributable improvements in household resilience may coincide with decreases in women’s empowerment outcomes around household decision-making and control of productive assets. Finally, the most vulnerable women in a community may be excluded from or forced out of groups due to negative perceptions of the poor ( Hossain & Rahman, 2018). These findings caution against the idea of interventions with women’s groups as sufficient to equitably support member resilience without addressing structural inequities.

***How do women’s groups support community responses to shocks?*** We found consistent evidence of women’s groups playing a role in the community response to a shock. The literature revealed diverse examples of women’s groups partnering with a broad range of stakeholders to support all stages of responses to various shocks. In particular, women’s groups had the ability to organize and empower women to advocate for more inclusive resilience policies that are better aligned with women’s priorities ( Clissold *et al.,* 2020), highlighting their potential role in transformative resilience through systemic change.

**Figure 1.**
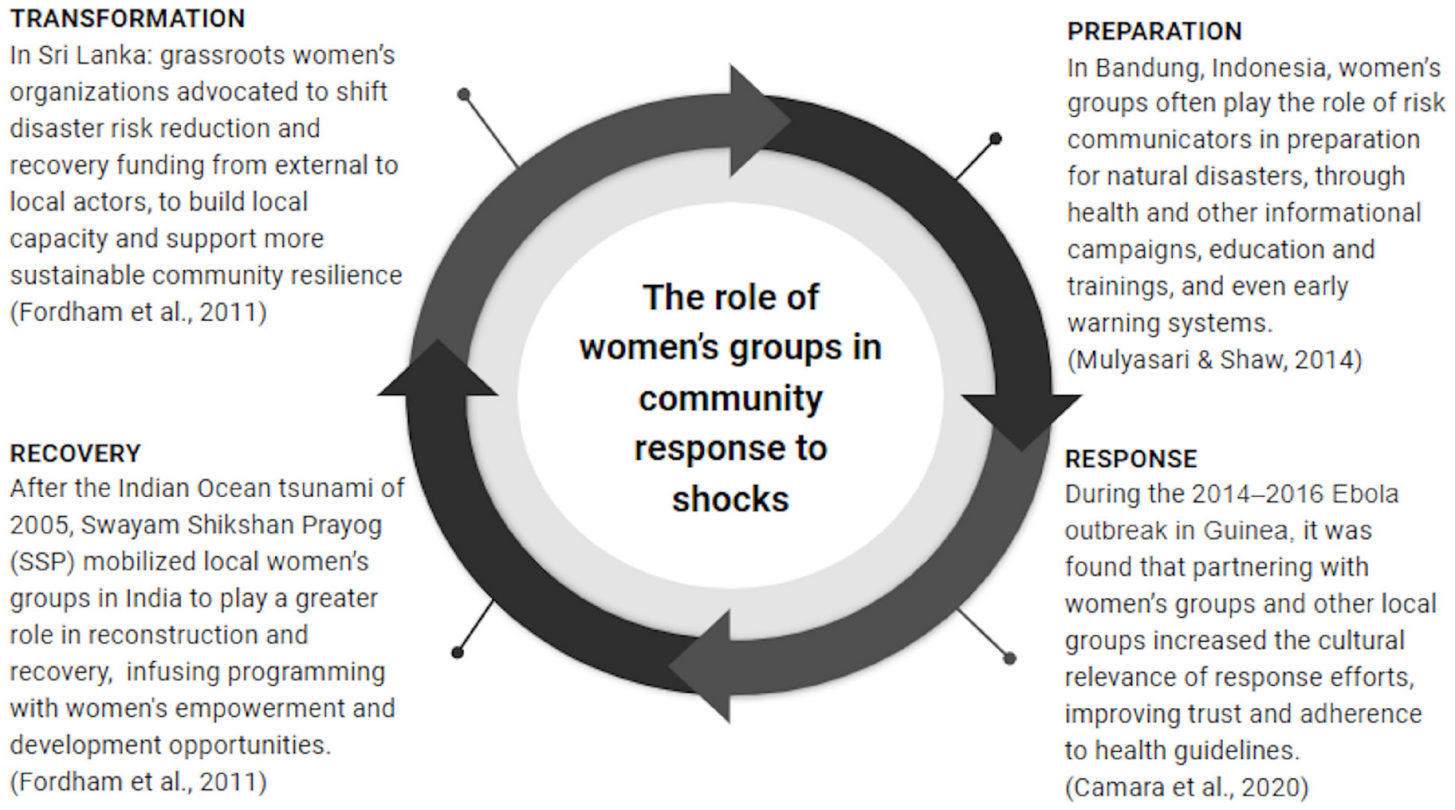
The role of women’s groups in the stages of community response to shocks.

Women’s groups have supported multiple stages of community response to a shock: from preparation and disaster risk reduction to immediate response and relief efforts, to reconstruction and recovery, and finally by advocating for transformational change (see Figure 1). This includes both pre-existing women’s groups, which often serve as valuable human infrastructure in the face of a shock, as well as new women’s groups that form to mobilize women to respond to a shock in their community. After large natural disasters in South Asia, women’s groups actively participated to rebuild and rehabilitate their community – by setting up relief camps ( Nambiar, 2016), taking charge of large community kitchens for the displaced ( Shaji, 2020), holding household cleaning drives after floods ( Anandan, 2018), providing psychological counselling to the bereaved ( Anandan, 2018), and contributing significant sums of money from their savings toward the rebuilding of their community infrastructure ( Anandan, 2018). We found that many types of local organizations that center women, inclusive of small lending groups as well as large, networked advocacy organizations, engage with a broad range of stakeholders in these community efforts, such as forming advocacy coalitions with women’s rights organizations ( Fisher, 2009; Fordham *et al.,* 2011), serving as government partners and implementers ( Yonder *et al.,* 2005), partnering with local and international NGOs (INGO) ( Fordham *et al.,* 2011) and working with multilateral institutions such as the UN ( Brickman Raredon, 2011).

The literature cites several locally relevant communication, implementation, monitoring, and advocacy roles that women’s groups can play in community responses to shocks. Women’s group connections to their communities mean that they “are talented in gathering local information that is difficult, if not impossible for outsiders to access” ( Yonder *et al.*, 2005, p. 35). Involving women’s groups in community responses can also increase the cultural relevance of programming, bolstering community trust and leading to an improved response ( Camara *et al.,* 2020). In their role as implementers of shock response programming, they can “function as intermediaries between their communities and the government in a manner that improves the speed, quality, and accountability of the government programs” ( Yonder *et al.,* 2005, p. 35). They can also support monitoring of a response to a shock – including everything from tracking incidents of gender-based violence in shelters ( Fisher, 2009), to outbreak surveillance and reporting ( Deepa *et al.,* 2008), to ensuring resources are allocated appropriately to those most in need ( Yonder *et al.,* 2005). Finally, grassroots women’s organizations appear to play an important advocacy role in ensuring government and INGO programming best supports women and their communities ( Fordham *et al.,* 2011).

However, women’s advocacy organizations, grassroots cooperatives, and federations of savings and credit groups have reported a lack of meaningful involvement in national disaster responses. Across 21 interviews of women’s advocacy organizations in Latin America, South Asia, and sub-Sharan Africa, Oxfam found that the majority felt “sidelined” from disaster response and recovery initiatives due to “perceived lack of technical capacity and reach”, as they “had never been consulted by aid agencies on resilience and disaster preparedness strategies, and had no contact with government and nongovernmental actors in charge of disaster response and recovery initiatives” ( Ravon, 2014, p. 17). Other sources corroborate this finding, as Goetz and Jenkins (2016) found multiple instances of the UN excluding women’s groups in the conflict resolution and peacebuilding processes, and Enarson, Fothergill, and Peek (2007) found that women and women’s grassroots organizations are often not in positions of authority during disaster relief and recovery. Gupta and Leung stated that “successful partnerships between grassroots women’s organizations and government agencies… are exceptions rather than the norm” in disaster risk reduction and recovery, and that typical programs such as aid and training too often “reproduce rather than redress women’s marginalization and vulnerabilities” (2011, p. 25). 

As a result, these and other authors call for more meaningful partnerships with, and investments in, organizations that work with women, which include community-based small groups such as VLSAs, networks or federations of women’s groups, and NGOs/INGOs that work with women’s networks and women’s rights/advocacy organizations. Experts cite the potential of such partnerships to reduce the perpetuation of gender inequity through building local capacity instead of relying upon external expertise ( Gupta & Leung, 2011), and by supporting the ability of local women to be “permanent active agents of resilient development” ( Fordham *et al.,* 2011, p. 65). Thus, our findings suggest that there is room for greater inclusion of women’s groups in community, government, and NGO response to shocks – and that increased inclusion has the potential to support community resilience.

***Spotlight on women’s groups and Ebola*.** The Ebola epidemic from 2014–2015 had a catastrophic impact on VSLAs in Liberia, including absenteeism for VSLA meetings and activities, a decrease in contributions and resultant reduced funds available for loans, and–ultimately–the suspension of all VSLA activities ( Langlay, 2014). Men who were able to borrow money in Liberia during the crisis mostly did so through informal sources, such as family and friends, while women primarily relied on credit offered by savings clubs and *susu clubs*, as other formal financial services were suspended ( Korkoyah & Wreh, 2015). In Sierra Leone, the burden of the Ebola shock decreased group members’ ability and propensity to contribute savings – which, in turn, put pressure on the group’s social fund, despite it being needed for expenses related to burial and the care of orphaned and vulnerable children ( Androsik, 2020). However, while the Ebola epidemic highlighted the vulnerability of women’s savings groups to shocks, it also showed their resilience – as many VSLA group members in Liberia remained steadfast in their commitment to their group throughout the crisis ( Langlay, 2014).

Women’s groups adapted their activities, services, and roles in response to Ebola outbreaks in various ways. In Liberia, the financial savings and loan activities of VSLAs were disrupted, but groups naturally evolved to serve as important sources of psychosocial support to members during this period of extreme loss and grief ( Langlay, 2014). In response to recurrent conflict and the Ebola crisis in the Democratic Republic of the Congo, VSLAs experimented with a “resilient VSLA model” to better cope with recurrent shocks that included shorter loan cycles, new emergency funds, and facilitating membership for displaced women ( CARE, 2020a). Women’s groups also adapted to play a role in the community response to Ebola in the DRC, where the World Health Organization trained women’s group representatives to spread awareness and share information in 30 Beni neighborhoods about vaccines, contact tracing, treatment, and the vulnerability of women and children to the disease ( WHO, 2018).

We found one study that incorporated a causal identification strategy to measure member resilience attributable to group membership during the Ebola outbreak. The study focused on the effectiveness of the Empowerment and Livelihood for Adolescents (ELA) intervention using a cluster-randomized controlled trial in Sierra Leone ( Bandiera *et al.,* 2019). The Ebola outbreak occurred in the midst of the intervention and varied in severity across intervention locations. Thus, the authors were able to measure the mitigating impact of belonging to an ELA group during Ebola. The study found that group membership decreased the amount of time adolescent girls spent with men, decreased out-of-wedlock pregnancy by 7.5 percentage points, and increased school attendance by 8.5 percentage points. In a follow-up analysis, the positive effects persisted in higher human capital accumulation (increased school enrolment at higher levels of education) over time for group members ( Bandiera *et al.,* 2020).

## Discussion

Several common themes emerged in our evidence synthesis of the relationship between women’s groups and acute covariate shocks. First, covariate shocks tend to disrupt group activities – either through reducing membership and meetings or by altering group functions to respond to the shock. Widespread shocks also tend to deplete group resources in a time of high need. However, linkages to formal institutions can mitigate the impact of shocks on group resources by extending access to credit beyond the shock-affected resource pool. Studies conducted in India with networked and institutionally linked SHGs were more likely to report positive resilience outcomes due to uninterrupted access to credit, while studies of more autonomous women’s groups tended to describe resource shortages and reliance on savings. Groups may adapt their policies during a shock by introducing more flexibility around contributions and loan repayments; however, the resulting benefits to members may come at the cost of group sustainability. Indeed, there may be a tension between the prioritization of group resilience versus individual resilience that is underexplored in the literature. Groups may also employ technical adaptations to promote resilience during shocks, such as switching to digital technology when in-person meetings are disrupted, but we did not find sufficient evidence of these types of technical adaptations in our review.

The evidence of women’s groups providing resilience to members is limited in quantity and scope but rich in content. Published studies prioritize economic SHGs experiencing weather shocks, but a few articles examine the social support mechanisms of groups amidst the psychological toll of conflict shocks. Results vary according to shock severity, group purpose and structure, and outcome measures, but the evidence is largely supportive of the ability of women’s groups to benefit members during covariate shocks. While evidence on specific mechanisms is limited, the literature suggests that accumulated savings, regular contributions, and flexible credit conditions may contribute to the ability of groups to mitigate the negative economic consequences of shocks for individuals, though with potentially differential consequences across members. However, the lack of subgroup analyses, such as differential impacts on members according to income or caste, is a prominent gap in the current literature.

The literature cites numerous benefits of women’s group involvement in the community response to a shock, but also suggests that women’s groups and advocacy organizations often feel sidelined. Women’s groups can provide valuable local expertise, human infrastructure, and community connections, with the potential to support governments and a variety of private actors in stronger, more sustainable responses to covariate shocks in contexts with wide coverage. Future research could expand upon this point and explore to what extent this engagement places a greater relative burden or risk on women *versus* to what extent it promotes women’s empowerment and contributes to shifting gender norms in communities.

## Conclusions

This review reveals that while women’s groups may provide resilience to members and communities, covariate shocks tend to disrupt group activities and reduce group resources. The findings thus suggest a trade-off between individual or household and group resilience, indicating that groups may require additional financial support to remain sustainable during and in the aftermath of crises. This observation is consistent with the finding that access to formal institutions can mitigate the negative impacts of shocks on group resilience. We found this tension between individual and group resilience during the Ebola crisis in Sierra Leone, as limited contributions by group members put pressure on the group’s social fund ( Androsik, 2020). However, evidence from Liberia shows that group members continued contributing to their group throughout the Ebola crisis, suggesting that the tradeoff between group and individual resilience may not apply in all contexts. 

The findings of this evidence review on the ability of group membership to promote resilience are consistent with early findings related to the short-term effects of COVID-19 on women’s groups and their members. Evidence from nationally representative longitudinal panel data based on in-person (before COVID-19) and phone-based (after COVID-19) surveys in Nigeria showed that households with a female member in a savings group experienced smaller increases in food insecurity than households without members ( Adegbite *et al.,* 2022). A recent study from Uganda found that membership in savings groups was associated with a lower likelihood of suffering income shocks and a lower likelihood of a reduction in food consumption ( Kansiime *et al.,* 2021). A study from India found mixed results, suggesting smaller decreases in consumption after COVID-19 for SHG members compared to non-members but no evidence for statistically significant associations with other economic outcomes ( de Hoop *et al.,* 2021). In addition, most women’s group farmers in Kerala, India, were able to get a fair return because they had access to enough intra-group labor to continue harvesting during COVID-19 ( Agarwal, 2021). Findings from India further indicate that SHGs faced challenges due to COVID-19 lockdowns, particularly lower mobilization of monthly savings, which may create challenges for group sustainability ( Siwach *et al.*, 2023). The study also showed that SHGs in geographies that received disbursements from the government experienced lower reductions in savings than SHGs in geographies without disbursement, which may have supported group resilience. VSLAs in multiple countries in sub-Saharan Africa also adapted their programming by introducing digital meetings, changing meeting frequency, and meeting with fewer members at a time after the gradual relaxation of the lockdown ( Adegbite *et al.,* 2022). Like women’s groups in Liberia during the 2014 Ebola outbreak, SHG members in India and savings group members across sub-Saharan Africa became involved in the community response to COVID-19 by partnering with government agencies to produce personal protective equipment (PPE), hand sanitizer, and masks and support vulnerable community members ( Adegbite *et al.,* 2022; Government of India, 2020).

While evidence is emerging about the initial implications of COVID-19 for women’s groups ( Adegbite *et al.,* 2022; Mulyampiti *et al.,* 2023), the long-term impacts of the pandemic on women’s groups and their members remain unknown. Future research can address this evidence-gap through mixed-methods research, exploring the ways in which women’s group membership may contribute to the long-term resilience of members and assessing the longer-term effects of covariate shocks on group sustainability (paying special attention to the role of external financial assistance to groups).

In the short term, we nonetheless have several take-aways from the evidence synthesis that may inform policy:

- **Sustainable access to financial and other resources, for both women’s groups and their members, is a crucial resilience mechanism to support women’s groups and their members during and in the aftermath of covariate shocks, such as COVID-19.** Uninterrupted access to credit with flexible conditions is important for member resilience, and support for women’s groups may include external contributions to group resources, such as cash transfers, to help members’ smooth consumption while normal income generating activities are suspended ( Tankha, 2020). For example, the Reserve Bank of India provided an option for one-time restructuring to borrowers in August 2020 in response to the pandemic. This option included SHG loans for which the account was classified as ‘standard’ as of March 1, 2020, and where defaults were not over 30 days. However, reports suggest that by the deadline of 31 December 2020, banks had received restructuring requests for only 2% of the loan book ( de Hoop *et al.,* 2021). Policy seeking to bolster resilience will likely need to go beyond the provision of resources for basic needs and instead incorporate a multidimensional approach that includes psychosocial support, access to health information, and protections against the increased risks of gender-based and domestic violence ( Tankha, 2020).
- **Meaningful partnerships with women’s groups during a community response to a shock have great potential.** Research suggests that including women’s groups in a meaningful way can produce benefits for the community as a whole and, especially where groups exist on a wide scale, for women’s overall resilience to shocks. Qualitative findings from Nigeria indicate that women’s groups may have helped to provide support to members in acute need, connecting women with new income opportunities and contributing to reductions in gender-based violence ( Agene & Onyishi, 2020; Mulyampiti *et al.,* 2023). However, evidence from India also shows that women producing masks in response to COVID-19 suffered due to delayed payments for goods procured on credit ( Kudumbashree State Mission, 2020), indicating that partnerships with women’s groups during crisis must also ensure risk mitigation and protection for women – such as ensuring timely payment ( CARE, 2020b). It also remains critical to ensure social distancing and access to PPE for women leaders and contributors in the response to COVID-19.
- **Policies and adaptations of interventions can have heightened potential for unintended adverse consequences during shocks.** Policymakers concerned with equity will need to consider—and monitor—the possibility that the benefits of women’s groups may not be distributed equally among members, and that the most vulnerable may be excluded from or harmed from groups during shocks. For example, the use of digital meetings has created barriers to participation for some of the most marginalized women ( Adegbite *et al.,* 2022). Care will also be necessary to ensure that interventions targeted toward women’s groups during shocks are carried out in an empowering way rather than increasing existing gendered burdens or perpetuating gender inequities.

The included studies provide a broad and comprehensive overview of the relationships between women’s groups and covariate shocks. However, as this review was rapid and not fully systematic, we want to acknowledge several limitations. Though we conducted extensive searches of various platforms, it is likely that we missed some of the evidence. Additionally, the research design and methodological rigor of the reviewed studies varied widely, and we did not conduct a risk of bias assessment on the included studies ( Hombrados & Waddington, 2012). Due to our broad scope, findings for one women’s group may have limited generalizability to other women’s groups across contexts or population groups ( *e.g.,* adolescents and adult women), and comparisons and effect aggregation across studies are challenging due to diverse outcome measurement, shock severity, and group type. In addition, detailed information about each women’s group (including gender composition) was often inconsistently specified. Finally, we were not able to include analyses on COVID-19 and women’s groups during the most recent COVID-19 humanitarian crises in India and Uganda because of the limited availability of evidence during our search timeframe.

Despite these limitations, reviewing the recent historical record on women’s groups and shocks was important to synthesize lessons learned and enable the generation of a broad evidence base on this topic. The review provides meaningful evidence for policymakers and practitioners engaged with women’s groups who aim to strengthen their long-term resilience after COVID-19. It also points to the ongoing importance of studying and documenting the relationship between women’s groups and serious but possibly more localized shocks that do not garner the research attention of COVID-19. When and where these shocks will take place is unpredictable, making rigorous study designs difficult; however, covariate shocks will continue to occur and will most likely disproportionately affect women in some of the world’s most vulnerable populations. A better understanding of women’s groups in the context of shocks may support pre-emptive policy and group action and hasten post-shock responses. Further research can also expand our understanding of how, and under what conditions, joining a group can support individual resilience and contribute to community responses.

## Data Availability

No data are associated with this article.

## Footnotes

^1^ Covariate shocks are shocks that occur at the community or macro-level (e.g., natural disasters, disease outbreaks, conflict, and economic crises) and may affect many households simultaneously.

^2^ The categories are mutually exclusive, but we did allow for multiple categories under organizing purpose. In case of doubt, we selected the category that was most closely aligned with the paper. However, the sources included in our search did not always fall neatly into categories, therefore there may have been some inconsistency in coding across the categories described.
