# Supplementary material for: Women's groups, covariate shocks, and resilience: An evidence synthesis of past shocks to inform a response to COVID-19
Source: Gates Open Res. 2023 Jul 20;7:111. [Version 1] doi: 10.12688/gatesopenres.14771.1 (PMC10442478; doi:10.12688/gatesopenres.14771.1)
Supplement: Peer review history

# Peer review: Reviewer response for version 1

**DOI:** 10.21956/gatesopenres.16097.r34361

**Published:** 2023-08-21

**Authors:** Manishree Mondal

**Affiliations:** 1 Department of Geography (UG & PG), Midnapore College, Midnapore, West Bengal, India

This article is an excellent qualitative meta-analysis regarding the activities of women’s groups in lower and lower middle countries of the world towards the survival and resilience of their members, community and society in the time of acute crisis of various covariate shocks prior to COVID-19. Both physical and socio-cultural shocks were discussed with proper illustrations.

 The major observations:

1. The abstract is well framed. Three basic questions are methodically analyzed with proper examples and elaborated descriptions. Empirical studies of twenty (20) quality documents are presented systematically and categorically in tabular format. Policy recommendations and sustainable pathways for the mitigation of problems faced by the women’s groups in the time of crisis as well as resilience or survival are brilliantly presented in the conclusion section. Limitations of this study are also mentioned clearly.
2. Objectives of the study are stated but not in a clear category. It may be troublesome for readers to find out easily.
3. Among the discussed 90 covariate shocks about 43 are related to climatic phenomena followed by 14 health related issues, 12 socio-cultural, 6 economic shocks and 15 tectonic disasters. Every shock has different devastating nature with differential impacts on society. It might be better to discuss these various types of shocks categorically.
4. Thrust has been given on only women’s group activities and resilience rather than the individual members but it was evident that every member of every unique group has different needs for their resilience and survival.
5. About 90 documents were extracted from 2800 search results. This is really a huge amount of literature review. It will be better if there was a table for searched literatures according to their quality of publications.
6. Qualitative analysis of the searched result is truly commendable but there is no quantitative or sensitivity analysis on the basis of their research quality. This qualitative analysis only presents the description of the scenario. The valid, objective based scientific analytical tools and techniques can produce high level of validate and reliable results. Randomized Controlled Trials (RCT’s) is one of them. Graphical presentation is more accountable rather than wordy explanations.
7. Publication bias is not mentioned in the method section. Due to the higher likeliness of publications of qualitatively both significant and non-significant studies, this biasness can distort the result.
8. The main aim of this article was the search of past experience of various women’s group against the different covariate shocks. The impact analysis of COVID-19 in the conclusion section may be little bit of puzzling to the readers. Actually COVID-19 is extremely an exceptional phenomenon with various facets which shattered the whole world. Even more literature analysis is necessary to draw any generalization on the impact of this incident on women’s group or their members.
9. Lastly, if two major facts finding tables are showed as supplementary files in appendices then the consistency of the whole analysis will be maintained more clearly.

This is a very contemporary and necessary topic of discussion. Women’s groups along with their members are really the most vulnerable in any kind of shock whether it is covariate or not. This type of analysis can able to show the right choice for resilience and survival against any type of risk and vulnerability.

Is the work clearly and accurately presented and does it cite the current literature?

Yes

If applicable, is the statistical analysis and its interpretation appropriate?

Not applicable

Are all the source data underlying the results available to ensure full reproducibility?

Yes

Is the study design appropriate and is the work technically sound?

Partly

Are the conclusions drawn adequately supported by the results?

Yes

Are sufficient details of methods and analysis provided to allow replication by others?

Yes

Reviewer Expertise:

Women's studies, social, cultural, environmental science, indigenous people.

I confirm that I have read this submission and believe that I have an appropriate level of expertise to confirm that it is of an acceptable scientific standard.

# Peer review: Reviewer response for version 1

**DOI:** 10.21956/gatesopenres.16097.r34359

**Published:** 2023-08-03

**Authors:** Timothée Demont

**Affiliations:** 1 CNRS, AMSE, Aix-Marseille University, Marseille, France

The article is a literature review, which brings no new results. This report is therefore purposely limited and focuses on the format. It gives advice to enhance the usefulness and the knowledge provided by this literature synthesis. I do think that the article represents a useful synthesis of the literature, in that it is very thorough and it tries to deliver a policy message at the end.

 My main comments are given below.

1. The overall readability could be improved. First, I would advise to focus on a limited number of clear and ordered results (focusing on strong evidence), clearly articulated in a logical framework (such as a theory of change distinguishing needs, mechanisms, intermediate and final outcomes). Details, as well as anecdotal and more fragile evidence could be relegated to a final section or an appendix. Second, the synthesis is very wordy and I would encourage the authors to use more graphical representations to show more visibly the nature, magnitude and dispersion of the main effects estimated by the literature.
2. Limit reference to gray literature and make a distinction between papers published in good peer-reviewed journals and papers unpublished or published in low-rank journals or that are purely qualitative and not representative of the population of interest. One possibility is to specify different confidence levels for each (family of) result, in the spirit of what IPCC does on climate. The readers need to be able to navigate easily through the massive number of studies, first focusing on strong evidence and then subsequently going through more tentative evidence if they are interested. It would also improve the readability of the literature synthesis.
3. I would also encourage the authors to use some more advanced meta-analysis tools, at least for part of the synthesis, e.g. discuss which percentage of the studies found positive, null, or negative effects, what is the average magnitude and its dispersion (variance). Of course, it would then become important to focus only on well-identified quantitative studies, and to recover average treatment effects from the different studies (taking into account that some report ATT, ITT, etc.). Again, the idea is to increase the actual knowledge generated by this literature synthesis.
4. Clarify if you consider having incorporated the universe of studies meeting your criteria or only a sample. In the latter case, specify the selection criteria.
5. Why didn’t you search the IDEAS ( https://ideas.repec.org/) database, which seems to be (one of) the first source(s) of papers in Economics?
6. Different versions of the same paper are sometimes quoted as if they were different papers. Prioritize the latest, ideally published, version.
7. The focus on trying to derive conclusions for the COVID-19 crisis is a bit odd. Given that it is a past event, it seems to me that there are two options. Either the authors focus on studies whose object was actually the response to COVID-19. Or the authors should rather present the main object of their study as being covariate shocks in general, of which pandemics are one example. And the objective should be to derive predictions regarding future covariate shocks, instead of a past event. Discussing the possibility to cope with covariate shocks is particularly important and interesting in the context of climate change (which is arguably a more crucial and pressing topic than pandemics). An additional issue is that shocks like COVID-19 implied a complete blockage of the society and the economy for some time, which is not true for most other covariate shocks such as weather shocks. Which complicates further this exercise of studying the literature on covariate shocks to derive conclusions for COVID-19. I would encourage the authors to change focus, with COVID-19 being one focus as they do for Ebola.
8. The difference between adaptive capacity and transformative capacity is not clear, both conceptually and empirically.
9. I would distinguish between (and compare) the very different types of women’s groups (in particular those with a focus on savings and credit, those with active NGO involvement, those involving specific profiles such as young girls or victims of violence). Trying to pool all models together actually decreases the informativeness of the report.
10. Effect on groups: distinguish short-term and long-term effects (some adaptation strategies, e.g. increased flexibility, can be good in the short term but detrimental to long-term group survival).
11. Discuss more the individual heterogeneity of the effects: we know it is strong, and it is very important to understand who is more and less vulnerable in a population after a covariate shock.
12. Can you say something about the externalities that women’s groups generate on local communities / GE effects?
13. I feel the discussion and conclusion are a bit disconnected with the literature review, focusing on points which are not the main ones. One (useful) thing is to explain what in your opinion is missing and/or what can or cannot be generalized etc. But when it comes to summarizing the findings from the literature, the authors should be very clear and fair with what the literature actually establishes (and what it does not), and, once again, try to make sense of it and extract a general knowledge (see comments 1, 2, 3).

Is the work clearly and accurately presented and does it cite the current literature?

Partly

If applicable, is the statistical analysis and its interpretation appropriate?

Not applicable

Are all the source data underlying the results available to ensure full reproducibility?

Yes

Is the study design appropriate and is the work technically sound?

Partly

Are the conclusions drawn adequately supported by the results?

Partly

Are sufficient details of methods and analysis provided to allow replication by others?

Yes

Reviewer Expertise:

Development economics

I confirm that I have read this submission and believe that I have an appropriate level of expertise to confirm that it is of an acceptable scientific standard, however I have significant reservations, as outlined above.

# Peer review: Reviewer response for version 1

**DOI:** 10.21956/gatesopenres.16097.r34360

**Published:** 2023-08-03

**Authors:** Siti Nurbayani, Moh. Dede

**Affiliations:** 1 Faculty of Social Sciences Education (FPIPS), Universitas Pendidikan Indonesia, Bandung, West Java, Indonesia; 2 Universitas Padjadjaran, Bandung, West Java, Indonesia

Thank you for this opportunity, your article is great. This review article summarizes new insiders about women and the global COVID-19 pandemic. Even so, there are several things that the authors must look at again hence your article is more interesting and makes it easier for readers to understand the content. Please look into the details.

 Kind regards,

 Siti Nurbayani & Moh. Dede

 Abstract:

- Research objectives have not been clearly presented.
- In terms of method, it has not been stated how to get published articles which are the basis for writing, especially how to find them.
- Conclusion it seems that there is no narrowing of the main findings.

Introduction:

- Too long section, could have been made more concise.
- Why is the sub-section “Women's groups and resilience” in this section? This should be put together with other paragraphs and make it coherent.
- Research objectives have not been clearly revealed.

Methods:

- Does this research use the SLR framework? If yes, please explain properly.
- Explain the procedure for this sentence “Our evidence synthesis is derived from academic databases, organizational reports, and additional gray literature. Using a targeted search strategy, we extracted 90 total documents from over 2,800 search results. It would be better if you included an illustration for this process.
- Why is the research question in this section? It should be sufficient to express it in a sentence, not a question, but a statement about the variables/parameters that you want to reveal.

Findings:

- Outstanding

Discussion:

- The author can make a kind of illustration as the synthesis in this section.
- Discussions and conclusions seem to overlap, this is quite confusing.

Conclusions:

- Some parts of this section should be in the discussion.
- This section should be made more concise.

Is the work clearly and accurately presented and does it cite the current literature?

Yes

If applicable, is the statistical analysis and its interpretation appropriate?

Not applicable

Are all the source data underlying the results available to ensure full reproducibility?

Yes

Is the study design appropriate and is the work technically sound?

Partly

Are the conclusions drawn adequately supported by the results?

Yes

Are sufficient details of methods and analysis provided to allow replication by others?

Partly

Reviewer Expertise:

Social studies, sexual harassment, family and gender, local wisdom, sociology education, and development

We confirm that we have read this submission and believe that we have an appropriate level of expertise to confirm that it is of an acceptable scientific standard, however we have significant reservations, as outlined above.
